# Identification and In Silico Selection of Novel Antioxidant Peptides from Asian Swamp Eel Bone: Quantum Chemical Calculations, Molecular Docking, and Zebrafish Model Validation

**DOI:** 10.3390/antiox15091057

**Published:** 2026-08-24

**Authors:** Xiao Wang, Jianan Zhang, Bingjie Chen, Xinlu Wang, Khushwant S. Bhullar, Yan Yang, Hongru Liu, Lan Wang, Chenggang Cai, Wenzong Zhou

**Affiliations:** 1 Crop Breeding and Cultivation Research Institution, Research Center for Agricultural Products Preservation and Processing, Shanghai Academy of Agricultural Sciences, Shanghai 201403, China; wangxiao.0127@163.com (X.W.);; 2 Hunan Key Laboratory of Forestry Edible Sources Safety and Processing, College of Food Science and Engineering, Central South University of Forestry and Technology, Changsha 410004, China; 3 Department of Agricultural Food & Nutritional Science, University of Alberta, Edmonton, AB T6G 2P5, Canada; 4 Institute of Edible Fungi, Shanghai Academy of Agricultural Sciences, Key Laboratory of Edible Fungi Resources and Utilization (South), Ministry of Agriculture, National Engineering Research Center of Edible Fungi, Shanghai 201403, China; 5 Key Laboratory of Agricultural Products Cold Chain Logistics, Ministry of Agriculture and Rural Affairs, Institute of Agro-Products Processing and Nuclear agricultural Technology, Hubei Academy of Agricultural Sciences, Wuhan 430064, China; 6 College of Biological and Chemical Engineering, Zhejiang University of Science and Technology, Hangzhou 310023, China; 7 Key Laboratory of Integrated Rice–Fish Farming Ecosystem, Ministry of Agriculture and Rural Affairs, Shanghai Academy of Agricultural Sciences, Shanghai 201403, China

**Keywords:** Asian swamp eel bone, antioxidant peptides, in silico screening, quantum chemistry calculation, molecular docking, zebrafish model

## Abstract

Fourteen novel antioxidant peptides were screened from the enzymatic hydrolysates of Asian swamp eel bone (ASEB) through an in silico analysis. Their ABTS and ORAC radical scavenging capacities were 1.70–4.29-fold and 2.79–5.90-fold higher than those of Trolox, respectively. Additionally, two novel peptide sequences (NVGW and WALN) were identified. Quantum chemical calculations combined with active–-site methylation experiments demonstrated that hydrogen atoms on tryptophan’s indole nitrogen, tyrosine’s phenolic hydroxyl, and arginine’s guanidinium group play crucial roles in enhancing ABTS and ORAC activities. Molecular docking further showed stable binding of ASEB peptides to myeloperoxidase (MPO) through hydrogen bonds and electrostatic interactions. Further studies indicated that ASEB alleviated oxidative stress in zebrafish by effectively reducing ROS accumulation, restoring redox homeostasis, and modulating the expression of Keap1–Nrf2 pathway-related antioxidant genes. These results enhance our understanding of the antioxidant properties of ASEB-derived peptides and support the high-value utilization of animal byproducts.

## 1. Introduction

The excessive generation of free radicals within organisms is frequently linked to the induction of oxidative stress, which can result in damage to DNA, proteins, lipids, and other cellular constituents [1]. This mechanism is involved in the development and progression of various diseases, including cardiovascular diseases, neurodegenerative disorders, diabetes, and cancer [2]. As a result, the maintenance of redox balance is regarded as essential for preservation of physiological homeostasis and overall health [3]. Within food-related systems, oxidative reactions induced by free radicals are known to deteriorate product quality, leading to changes in nutritional value, flavor, and texture [4]. These adverse effects can be attenuated by antioxidants, which effectively neutralize reactive species and mitigate oxidative stress [5]. Compared to synthetic agents, natural antioxidants are preferred because of their low toxicity and minimal side effects [6]. Due to potent antioxidant activity, favorable biocompatibility, and high bioavailability, antioxidant peptides have attracted considerable attention for potential applications in functional foods, nutraceuticals, cosmetics, and pharmaceutical products [7,8,9].

In recent years, increasing attention has been paid to the use of protein-rich byproducts for producing antioxidant peptides. Fish bones, a major byproduct of aquatic processing, are rich in structural proteins including collagen and osteocalcin [10,11]. These proteins are recognized for their considerable potential for reuse and value-added development. Enzymatic hydrolysis has been employed to extract bioactive peptides from fish bones, which display various beneficial effects, such as antioxidant, anti-inflammatory, and antihypertensive activities [12,13]. This process not only promotes environmental protection and resource recycling but also provides natural, safe, and low-allergenicity feedstocks for producing functional foods, nutraceuticals, and cosmetic products [14]. It has been reported that Asian swamp eel (*Monopterus albus*) exhibits significant antioxidant potential attributable to its diverse protein composition, balanced amino acid profile, and superior nutritional characteristics [15,16]. Morphologically, it resembles the yellow eel (*Anguilla marmorate*), featuring a BV/TV (bone volume/total tissue volume) of 17.9% ± 3.9%, providing a useful reference for understanding its skeletal structure and overall body composition [17]. However, systematic investigations into the antioxidant properties of peptides originating from its bone proteins remain limited.

Chemically based antioxidant assays have been extensively employed because of their simple procedure, quick response, and sensitivity [18]. These methods enable rapid evaluation of antioxidant activity. However, they are limited to identifying specific active sites or regions, and typically do not account for physiological conditions, bioavailability, and metabolic processes [19]. Thus, elucidating the relationships between antioxidant activity and the structural and chemical features of antioxidants is essential for the rational design and development of effective antioxidant agents, particularly to improve their efficacy and bioavailability in biological systems [20]. Density functional theory has demonstrated that peptides with elevated highest occupied molecular orbital (E_HOMO_) energy levels tend to function as hydrogen donors, facilitating free radical scavenging and metal ion chelation [18]. Moreover, elongation of hydrogen bonds has been associated with reduced bond dissociation energy, which promotes proton donation and enhances antioxidant activity [18]. Quantum chemistry approaches thus provide mechanistic and predictive approaches for assessing the antioxidant properties of peptides and determination of active sites responsible for radical scavenging and metal ion chelation [21]. Myeloperoxidase (MPO), a major contributor to oxidative stress in organisms, has been targeted through molecular docking with antioxidant peptides to simulate potential inhibitory mechanisms. This approach allows for molecular-level validation of their potential as MPO inhibitors, supporting their antioxidant and anti-inflammatory bioactivities [22]. The zebrafish embryo model is widely used as an in vivo system for the evaluation of antioxidant activity in peptides because of its optical transparency, rapid development, and ease of manipulation [23]. Induction of oxidative stress allows real-time assessment of reactive oxygen species (ROS) scavenging and the tissue-protective effects of peptides, while toxicity and safety parameters are assessed simultaneously. In addition, its high-throughput, low cost, and visual accessibility make this model particularly suitable for antioxidant peptide screening and mechanistic studies [24]. Therefore, the integration of computational simulation and in vivo validation provides an effective strategy for improving antioxidant peptide screening efficiency and elucidating mechanisms of action, thereby facilitating the rational development of bioactive peptides [25].

This study aimed to identify novel antioxidant peptides from Asian swamp eel bone (ASEB) and elucidate their potential antioxidant mechanisms through integrated in vitro evaluation, computational analysis, and in vivo validation. ASEB was hydrolyzed using five proteases, and Alcalase-derived hydrolysates with high hydrolysis efficiency and antioxidant potential were selected for peptide fractionation, identification, and screening. Fourteen candidate antioxidant peptides were obtained through in silico prediction and further evaluated after chemical synthesis. Quantum chemical calculations and site-specific modification analysis were used to characterize key active sites, while molecular docking with MPO was performed to investigate molecular interactions. The antioxidant effects and regulatory mechanisms of ASEB-derived peptides were further assessed in a zebrafish embryo oxidative stress model. This study provides insights into the structure–activity relationships and antioxidant mechanisms of ASEB-derived peptides, supporting their potential application in functional foods and high-value utilization of animal byproducts.

## 2. Materials and Methods

### 2.1. Materials

ASEB (protein content approximately 25%) was obtained from the Hubei Yuntaifang Co., Ltd. (Xiantao, China), washed to remove impurities, dried, ground, and passed through a 40-mesh sieve. Neutrase, flavorzyme, alcalase, papain, and protease M were purchased from Beijing Dahonglihui Biotechnology Center (Beijing, China). 6-Hydroxy-2,5,7,8-tetramethylxanthen-2-carboxylic acid (Trolox) and 2,2′-azobis [2-methylpropionamide] dihydrochloride (AAPH) were obtained from Beijing Yishanhuitong Technology Co., Ltd. (Beijing, China). 2,2′-Azino-bis(3-ethylbenzothiazol-5-sulfonic acid) disodium salt (ABTS) and sodium fluorescein were obtained from Beijing Zhongcheng Jinnian Technology Co., Ltd. (Beijing, China). Sephadex G-25 was sourced from Shanghai Yuanye Biotechnology Co., Ltd. (Shanghai, China). Synthetic peptides and active site methylated peptides (including NVGW, WALN, DFLRY, GYLPR, WGKLD, YVDRF, RWPVDL, WDPDAR, WLDQPH, GPYLDLK, RDWPDAR, WGDLSPK, WGDLSRP, and WDAAPGVPR, all with purity exceeding 95%) were obtained from Nanjing JiePeptide Biotechnology Co., Ltd. (Nanjing, China). Lipopolysaccharide (LPS, catalog number SMB00610) was purchased from Sigma. 2′,7′-Dichlorofluorescin diacetate (DCFH-DA) and N-acetyl-L-cysteine (NAC) were obtained from Biyuntian Biotechnology Co., Ltd. (Shanghai, China). The bicinchoninic acid (BCA) total protein quantification kit, catalase (CAT) assay kit, malondialdehyde (MDA) assay kit, glutathione peroxidase (GSH-Px) assay kit, superoxide dismutase (SOD) enzyme activity assay kit, reduced glutathione (GSH) assay kit, oxidized glutathione (GSSG) assay kit and total antioxidant capacity (T-AOC) kit were acquired from Nanjing Jiancheng Haihao Biotechnology Co., Ltd. (Nanjing, China). All other reagents used in this experiment were of analytical grade and sourced from China.

### 2.2. Enzymatic Hydrolysis of ASEB

Based on previous laboratory studies, five different proteolytic enzymes were employed for the hydrolysis of ASEB powder: neutrase (pH 7.0, 50 °C), flavorzyme (pH 7.0, 55 °C), alcalase (pH 8.0, 55 °C), papain (pH 7.0, 55 °C), and protease M (pH 7.0, 55 °C) [26]. In each trial, 10 g of ASEB powder was suspended in 200 mL of distilled water, and the pH was regulated based on the optimal value for the selected enzyme. Enzymatic hydrolysis was initiated by adding the enzyme solution at 4% (*w*/*w*) ratio of substrate mass. The reaction was carried out under the corresponding pH and temperature conditions for 6 h. The reaction was terminated by incubation at 100 °C for 10 min, after which it was cooled to room temperature. Subsequently, the hydrolysate was centrifuged at 7000× *g* for 10 min, and the supernatant was collected and lyophilized for further analysis [26].

### 2.3. Determination of Degree of Hydrolysis

The degree of hydrolysis (DH) was determined with slight modifications to the method reported by Marinou et al. [27]. The o-phthalaldehyde (OPA) reagent was prepared by first dissolving o-phthalaldehyde (160.0 mg) in ethanol (4.0 mL). Subsequently, borax (7.62 g) and sodium dodecyl sulfate (SDS, 200.0 mg) were dissolved in deionized water (150 mL), followed by the addition of dithiothreitol (DTT, 176.0 mg). Finally, the mixture was adjusted to a total volume of 200 mL with deionized water.

For determination, 20 μL of sample solution was added to 150 μL of freshly prepared OPA reagent and allowed to react for exactly 2 min. Absorbance was subsequently measured at 340 nm (A_sample_). Deionized water and a 0.1 mg/mL serine solution were used as the blank (A_blank_) and reference standard (A_standard_), respectively. The free amino group content, expressed as serine equivalents, together with the DH, was then calculated accordingly.

$$Ser\;NH_{2}\;\left(\mathrm{mmol}/g\right)=\frac{A_{\mathrm{sample}}-A_{\mathrm{blank}}}{A_{\mathrm{standard}}-A_{\mathrm{blank}}}\times 0.9516\times C_{\mathrm{sample}}$$

$$DH(\%)=\frac{\mathrm{Ser}\;{\mathrm{NH}}_{2}-\beta }{\alpha h_{tot}}\times 100\%$$

In the equation, C_sample_ (g/L) denotes the protein concentration of the sample. The constants *α*, *β*, *h_tot_* were set to 1.0, 0.4 and 8.3 mmol/g, respectively. A serine solution at 0.1 mg/mL corresponded to a molar concentration of 0.9516 mM.

### 2.4. Quantification of Protein Recovery

Nitrogen content of both the hydrolysate and raw ASEB was measured using an elemental analyzer (Vario EL Cube, Elementar, Langenselbold, Germany) and subsequently converted to protein content. Protein recovery was expressed as the percentage of total protein recovered in the lyophilized hydrolysate relative to the protein originally present in the raw material [28].

### 2.5. Determination of Antioxidant Activities

#### 2.5.1. ABTS Radical Scavenging Activity

The ABTS radical scavenging capacity was measured with slight modifications to the method of Wang et al. [29]. The ABTS radical cation (ABTS•**^+^**) was generated by reacting equal volumes of 7 mM ABTS and 2.45 mM potassium persulfate in the dark for 12–16 h and diluted with 50 mM phosphate buffer (pH 7.4) to an absorbance of 0.700 ± 0.020 at 734 nm. The assay was performed by mixing 50 μL of sample with 150 μL of ABTS•**^+^** solution, followed by incubation at 30 °C for 30 min. Absorbance was measured at 734 nm. Results were expressed as Trolox equivalent antioxidant capacity (TEAC), with phosphate buffer and Trolox serving as the blank and standard, respectively.

#### 2.5.2. Oxygen Radical Absorption Capacity (ORAC) Assay

The ORAC assay was performed with slight modifications to the method of Wang et al. [30]. A mixture of 20 μL sample and 120 μL fluorescein (117 nM) was incubated at 37 °C for 15 min in the dark, followed by initiation with 60 μL of 40 mM AAPH. Fluorescence signals were recorded every minute for 100 min at excitation/emission wavelengths of 485/520 nm using a Thermo Fisher Scientific microplate reader (Waltham, MA, USA). All reagents were prepared in 75 mM phosphate buffer (pH 7.4), with Trolox used as the calibration standard. ORAC values were obtained from the area under the fluorescence decay curve and expressed as Trolox equivalents.

### 2.6. Purification, Characterization, and In Silico Screening of Antioxidant Peptides

#### 2.6.1. Purification of Antioxidant Peptides

Gel filtration chromatography using a Sephadex G–25 column (3.7 × 27.8 cm) was employed to fractionate the enzymatic hydrolysate. The column was pre–equilibrated with deionized water and eluted using the same solvent. Elution was tracked by monitoring absorbance at 214 nm and 254 nm. Fractions corresponding to identical peaks were pooled, lyophilized, and stored for further analysis. Fractions exhibiting stronger antioxidant activity were selected for subsequent studies [26].

#### 2.6.2. Peptide Identification

Peptide sequences were identified using a Nano-LC-MS/MS system consisting of an Easy-nLC 1200 coupled with a Q Exactive mass spectrometer (Thermo Fisher Scientific, Waltham, MA, USA). Peptides were separated on a C18 column (100 μm i.d. × 170 mm, Reprosil-Pur 120 C18-AQ, 3 μm) at 45 °C using a gradient elution with mobile phases A (0.1% formic acid in water) and B (80% acetonitrile containing 0.1% formic acid) for 66 min. MS analysis was performed in the positive ESI mode with a spray voltage of 3.2 kV and an ion transfer tube temperature of 320 °C. Data were acquired in data-dependent acquisition mode over a mass range of 300–1800 m/z. Full MS scans were acquired at a resolution of 70,000, while MS/MS scans were obtained at a resolution of 17,500. The 20 most intense precursor ions were sequentially selected for MS/MS analysis (Top20) with a collision energy of 28.

Raw MS data were analyzed using PEAKS Studio software (version 11.0, Bioinformatics Solutions Inc., Waterloo, ON, Canada) for de novo peptide identification. The mass tolerance for precursor and fragment ions was set to 20 ppm. Peptide sequences were inferred using the PEAKS de novo algorithm based on a dynamic programming approach, and the confidence of sequence assignments was evaluated using the Average Local Confidence (ALC) score (0–100%). Peptides with an ALC score ≥ 85% were considered high-confidence de novo identifications and selected for subsequent analysis [26].

#### 2.6.3. In Silico Screening of Antioxidant Peptides

Candidate antioxidant peptides were screened using a multi-step in silico approach. PeptideRanker was first used to predict peptide bioactivity, and sequences with scores ≥ 0.5 were retained for further analysis. The retained peptides were subsequently evaluated for allergenicity and toxicity using AllerTOP and ToxinPred, respectively [26]. BIOPEP analysis was performed to assess their association with previously reported antioxidant peptides [31]. In addition, amino acid composition was analyzed to identify peptides enriched in hydrophobic residues and antioxidant-related aromatic amino acids (Trp and Tyr). Based on these criteria, peptides with favorable bioactivity scores, safety profiles, and structural characteristics were selected for subsequent validation [26].

### 2.7. Identification of Antioxidant Peptide Active Sites via Density Functional Theory (DFT) Calculations

The initial three-dimensional structures of the candidate antioxidant peptides were generated using Chem 3D software (version 21.0, PerkinElmer Informatics, Waltham, MA, USA), followed by preliminary energy minimization to obtain reasonable starting conformations. Subsequently, geometry optimization was performed using density functional theory (DFT) at the M06-2X/6-31G(d) level with the Gaussian 09 software package. Vibrational frequency calculations were conducted at the same theoretical level to confirm that the optimized structures corresponded to local minima without imaginary frequencies [32]. Based on the optimized lowest-energy conformations, electronic structure parameters, including the highest occupied molecular orbital energy (E_HOMO_), HOMO orbital distribution, Mulliken atomic charges, and selected bond lengths, were calculated to evaluate the electronic properties and potential antioxidant activity of the peptides.

### 2.8. Molecular Docking of Antioxidant Peptides with MPO

Molecular docking was performed to investigate interactions between antioxidant peptides and MPO. The crystal structure of MPO (PDB ID: 3F9P) was obtained from the Protein Data Bank (https://www.rcsb.org/structure/3F9P, accessed on 15 August 2024). Prior to docking, water molecules were removed using PyMol, and hydrogen atoms and charges were added with MGLTools. Peptide three-dimensional structures were generated with ChemDraw 20.0 [26]. Docking simulations were carried out using AutoDock Tools (version 1.5.6, The Scripps Research Institute, La Jolla, CA, USA), and the conformation with the lowest binding energy was selected for further analysis. Interaction visualization was performed using PyMol and Discovery Studio 4.5 [33].

### 2.9. In Vivo Studies in Zebrafish

#### 2.9.1. Zebrafish Maintenance and Embryo Collection

Wild-type AB zebrafish were obtained from the National Zebrafish Resource Center (NZRC) and maintained at 28 ± 0.5 °C under a 14 h light/10 h dark cycle. Fish were fed Artemia nauplii twice per day, with the amount of feed adjusted according to the previous day’s consumption to ensure complete ingestion within 3–5 min [34]. Following a 2-week acclimation period, healthy and sexually mature zebrafish were selected and transferred to a 1:2 male-to-female ratio in breeding tanks. Fertilized embryos were collected at 10:00 on the subsequent day and rinsed three times with ultrapure water before being transferred to E3 medium (5 mM NaCl, 0.17 mM KCl, 0.33 mM CaCl_2_, and 0.33 mM MgSO_4_) [35]. All experiments complied with the ethical guidelines of the Laboratory Animal Welfare Ethics Committee of the Guangdong Provincial Human Microbiome Engineering Technology Research Center (Approval No.: IACUC MC 0811-01-2025).

#### 2.9.2. Toxicity Analysis of Antioxidant Peptides on Zebrafish Embryos

Normally developing zebrafish embryos at 8 h post-fertilization (hpf) were selected and randomly assigned to experimental groups. Embryos were seeded in 24-well plates at 10 embryos per well with three replicate wells per group. Each well was treated with 1 mL of antioxidant peptide solution at concentrations of 0, 50, 100, 200, 400, and 800 μM, representing six concentrations across 14 experimental groups [35]. All embryos were maintained at 28.5 °C in a constant-temperature incubator. Mortality and deformity rates were recorded and statistically analyzed at 24, 48, 72, 96, and 120 hpf [35].

#### 2.9.3. Detection of ROS Levels in Zebrafish Embryos

Three days post-fertilization (dpf) normally developed zebrafish embryos were randomly selected and distributed into a 48-well plate (10 embryos per well, one well per group). Four experimental groups were established: control (E3 solution), model (25 μg/mL LPS), positive control (250 μM NAC + 25 μg/mL LPS), and test (100 μM ASEB peptide + 25 μg/mL LPS). Each well was filled with 2 mL of the corresponding solution and incubated at 28.5 °C for 72 h, with solutions refreshed every 24 h. After incubation, solutions were removed, and embryos were washed three times with E3 medium. A 20 μg/mL DCFH-DA solution was then added, followed by incubation in the dark for 1 h. After washing three additional times, embryos were observed under an inverted fluorescence microscope (Leica Microsystems, Wetzlar, Germany) [35]. Fluorescence intensity was quantified using ImageJ (version 1.54, National Institutes of Health, Bethesda, MD, USA), and ROS levels were calculated as the percentage of fluorescence intensity relative to the blank group. Treated groups included the model, positive control, and ASEB peptide groups [24].

#### 2.9.4. Determination of MDA, GSH/GSSG, SOD, CAT, GSH-Px, and T-AOC in Zebrafish Embryos

Following the ROS level detection protocol, normally developing zebrafish embryos at 3 dpf were randomly selected. Embryos were placed in 6-well cell culture plates, with 40 embryos per well and 5 mL of solution added to each well. Three wells were established per group. Plates were incubated at 28.5 °C for 72 h, with the solution replaced every 24 h. After incubation, 20 larvae per group were randomly collected as samples for subsequent analyses. Following euthanasia, samples were homogenized in 600 μL of physiological saline, centrifuged at 4 °C (2500 rpm, 10 min), and the resulting supernatants were collected for analysis [34]. MDA levels and antioxidant enzyme activities, including SOD, CAT, GSH-Px, and T-AOC, were determined using commercial assay kits (Nanjing Jiancheng Haihao Biotechnology Co., Ltd., Nanjing, China) [35].

#### 2.9.5. Effects of Antioxidant Peptides on Keap1, Nrf2, HO-1, GCL, and GSS Gene Expression

Zebrafish were grouped as described above. Total RNA was extracted using the G3640-50T kit (Seville Biotechnology Co., Ltd., Wuhan, China), and cDNA was synthesized with the KR128 fastking one-step system (Beijing Tiangen Biochemical Technology Co., Ltd., Beijing, China) to remove genomic DNA [34]. Quantitative real-time PCR was carried out on a lightcycler 96 system (Roche, Basel, Switzerland). Gene expression was quantified using the 2^−ΔΔCt method, with actin mRNA serving as the internal reference for normalization [34]. The PCR program consisted of an initial denaturation step at 95 °C for 3 min, followed by 40 amplification cycles of denaturation at 95 °C and annealing at 60 °C. Primer sequences are provided in Table S1.

### 2.10. Statistical Analysis

Data were summarized as mean ± SEM from three to seven independent experiments. Differences among groups were analyzed by one-way ANOVA using GraphPad Prism 6 (GraphPad Software, San Diego, CA, USA), with Dunnett’s test applied for post hoc multiple comparisons versus the vehicle control. Statistical significance was set at *p* < 0.05, *p* < 0.01, and *p* < 0.001.

## 3. Results and Discussion

### 3.1. Preparation, Purification, and Identification of Antioxidant Peptides from ASEB

ASEB proteins were hydrolyzed enzymatically to produce antioxidant peptides, an effective approach for obtaining bioactive peptide fractions [36]. Since proteolytic specificity governs peptide profiles, the selection of protease markedly influences the molecular characteristics and antioxidant performance of the hydrolysates [37]. Among the five enzymes evaluated, alcalase yielded hydrolysates with superior protein recovery and antioxidant capacity (Figure S1, *p* < 0.05). The improved performance may be attributed to the partial unfolding of ASEB proteins under alkaline conditions, which increases enzyme accessibility. In addition, the broad substrate specificity of alcalase enables extensive peptide bond cleavage, favoring the generation of antioxidant peptides. The alcalase hydrolysate was subsequently purified by gel filtration chromatography (Figure S2), and fraction F4 exhibited the strongest antioxidant activity. UPLC–MS/MS analysis identified 526 peptides in F4 with confidence scores ≥ 85%.

**Figure 1:**
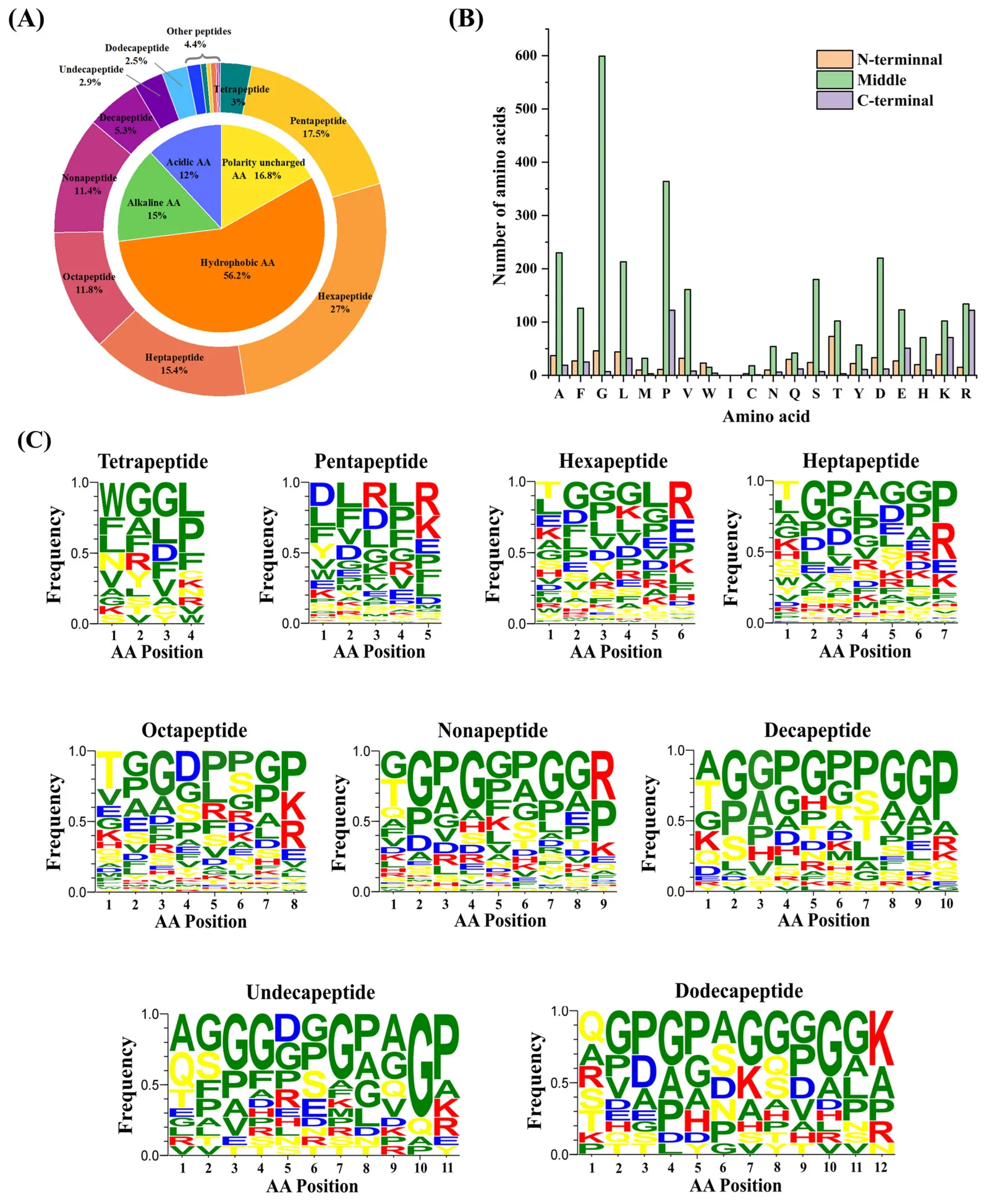
Comprehensive analysis of sequence features and amino acid composition of ASEB-derived peptides: (**A**) Classification of identified peptides based on amino acid physicochemical properties (inner ring) and peptide distribution (outer ring). (**B**) Distribution frequency of individual amino acid residues in identified peptides. (**C**) Relationship between peptide length and amino acid composition. In panel (**C**), amino acids are colored as follows: green, nonpolar amino acids; blue, acidic amino acids; red, basic amino acids; and yellow, polar amino acids.

As illustrated in Figure 1, peptides were mainly composed of 5–9 amino acid residues. Hexapeptides were the most abundant (26.42%), followed by pentapeptides (17.49%), heptapeptides (15.40%), nonapeptides (11.41%), and octapeptides (11.22%) (Figure 1A, outer ring). Hydrophobic amino acids were the most abundant, accounting for 56.23% of total residues, followed by uncharged polar (16.82%), basic (14.99%), and acidic (11.96%) residues (Figure 1A, inner ring). As illustrated in Figure 1B, all 20 standard amino acids were identified. Among them, glycine (G), proline (P), leucine (L), alanine (A), arginine (R), and aspartic acid (D) exhibited the highest frequencies, accounting for 16.74%, 12.76%, 7.42%, 7.34%, 6.96%, and 6.80% of total residues, respectively. Hydrophobic residues, particularly G, P, L, and A, are known to enhance antioxidant capacity, especially at the N– or C–terminus [38]. In this work, 43.73% of peptides possessed hydrophobic residues at the N–terminal localization, and 41.83% at the C–terminal end. SeqLogo analysis further revealed the distribution of amino acids across different peptide lengths (Figure 1C). Hydrophobic residues predominated at all lengths, followed by uncharged polar and basic amino acids. With increasing length (5–11 residues), the proportion of hydrophobic residues increased from 52.04% to 65.45%, whereas acidic and basic residues declined from 17.39% to 5.71% and from 18.91% to 9.09%, respectively. Notably, the C–terminal region of peptides containing 5–9 residues exhibited a high proportion of basic amino acids, with proportions exceeding 38.27%. These results indicate that the ASEB antioxidant peptides are generally short in length and enriched in hydrophobic and functional amino acids, supporting their pronounced antioxidant potential.

### 3.2. In Silico Screening and Antioxidant Verification

Antioxidant peptides were screened using PeptideRanker, a bioinformatics tool that predicts peptide bioactivity using sequence-derived features. Peptides scoring > 0.5 were classified as potentially bioactive [39]. From 539 candidate peptides, 45 peptides were selected according to the following criteria: PeptideRanker score > 0.5, absence of predicted allergenicity, and non-toxicity (Table S2). Aromatic residues, particularly tyrosine (Y) and tryptophan (W), are known to enhance antioxidant activity [9]. Their effects are further strengthened by hydrophobic residues, which can facilitate interactions with radical species [40]. Based on the high content of these residues, fourteen peptides (NVGW, WALN, DFLRY, GYLPR, WGKLD, YVDRF, RWPVDL, WDPDAR, WLDQPH, GPYLDLK, RDWPDAR, WGDLSPK, WGDLSRP, and WDAAPGVPR) were selected for chemical synthesis. Table 1 summarizes the synthesized peptides (4–9 residues) with hydrophobicity (−0.63–0.13), steric hindrance (0.47–0.71), and amphiphilicity (0 or 0.73).

Peptide antioxidant capacities were assessed using ABTS and ORAC assays. All fourteen peptides exhibited Trolox equivalent (TE) values exceeding 1.0 μmol TE/μmol, indicating higher activity than Trolox (Table 1). Sequence alignment via the NCBI database revealed that NVGW and WALN had not been previously reported, and their MS/MS spectra are shown in Figure S3. No identical sequences were found for any of the fourteen peptides in the BIOPEP database, thereby confirming their novelty as antioxidant peptides. Among these candidates, five peptides (WALN, GPYLDLK, PDWPDAR, WGDLSRP, and WDAAPGVPR) displayed notably higher antioxidant activities. Their ABTS radical scavenging values ranged from 3.02 ± 0.03 to 3.65 ± 0.02 μmol TE/μmol, whereas ORAC values ranged from 4.11 ± 0.11 to 5.90 ± 0.28 μmol TE/μmol. Compared with previously reported antioxidant peptides, the peptides identified in this study exhibited stronger antioxidant activities. For instance, the sea squirt-derived peptides WLP and ISW showed ORAC values of 2.72 ± 0.47 and 1.93 ± 0.01 μmol TE/μmol, respectively [41], while the sea bass-derived peptide EYGTVVVFQ exhibited ORAC and ABTS radical scavenging values of 1.43 and 2.34 μmol TE/μmol, respectively [42].

The enhanced activities are attributable to their amino acid compositions and sequence characteristics. Hydrophobic residues, including W, A, L, and P, were present in high abundance and are likely to promote interactions with radical species. Aromatic residues Y and W, owing to their hydrogen-donating and electron transfer capacities, are widely recognized as effective radical quenchers. Peptides such as WALN, PDWPDAR, and WDAAPGVPR contain these residues. Radical scavenging occurs through electron/hydrogen donation, leading to increased ABTS and ORAC signals. Furthermore, basic residues such as lysine (K) and arginine (R) may promote electrostatic interactions with negatively charged radicals or peroxides, further contributing to antioxidant capacity. WALN and NVGW are previously unreported tetrapeptides that exhibit several structural characteristics frequently observed in antioxidant peptides, including a short sequence length, low molecular weight, high hydrophobicity, and the presence of tryptophan residues, suggesting a favorable structural basis for their antioxidant activity. It was also hypothesized that the spatial arrangement and conformational flexibility of peptide structures may influence the accessibility of active sites, thereby affecting antioxidant performance. Although in silico predictions and in vitro chemical assays have been widely employed for preliminary screening, key aspects of antioxidant mechanisms such as active site identification, elucidation of the targeting mechanism, and theoretical guidance for structural optimization are not fully addressed by these approaches. Accordingly, further investigation is required to clarify structure–activity relationships and confirm the antioxidant mechanisms.

### 3.3. Quantum Chemistry Insight into Antioxidant Reaction Sites of ASEB Peptides

**Figure 2:**
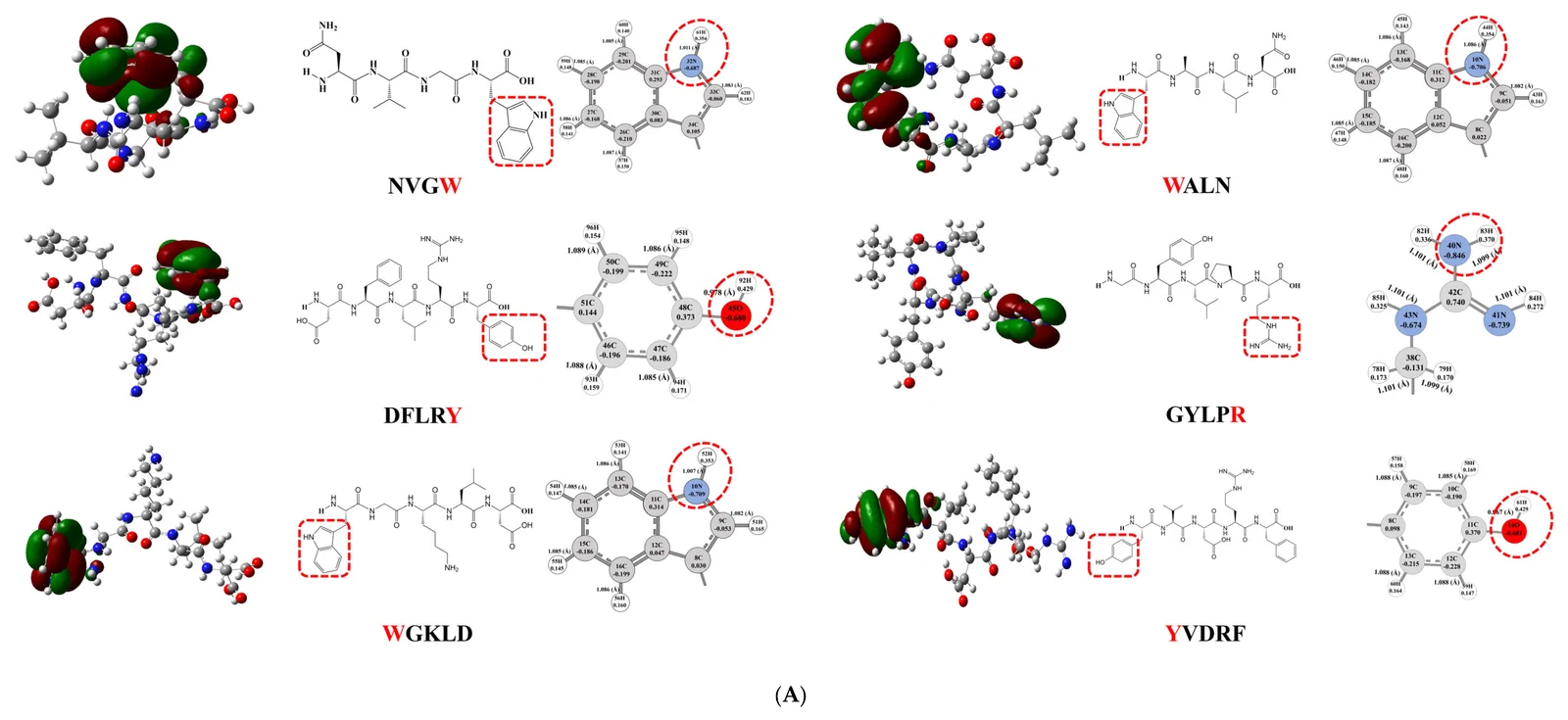

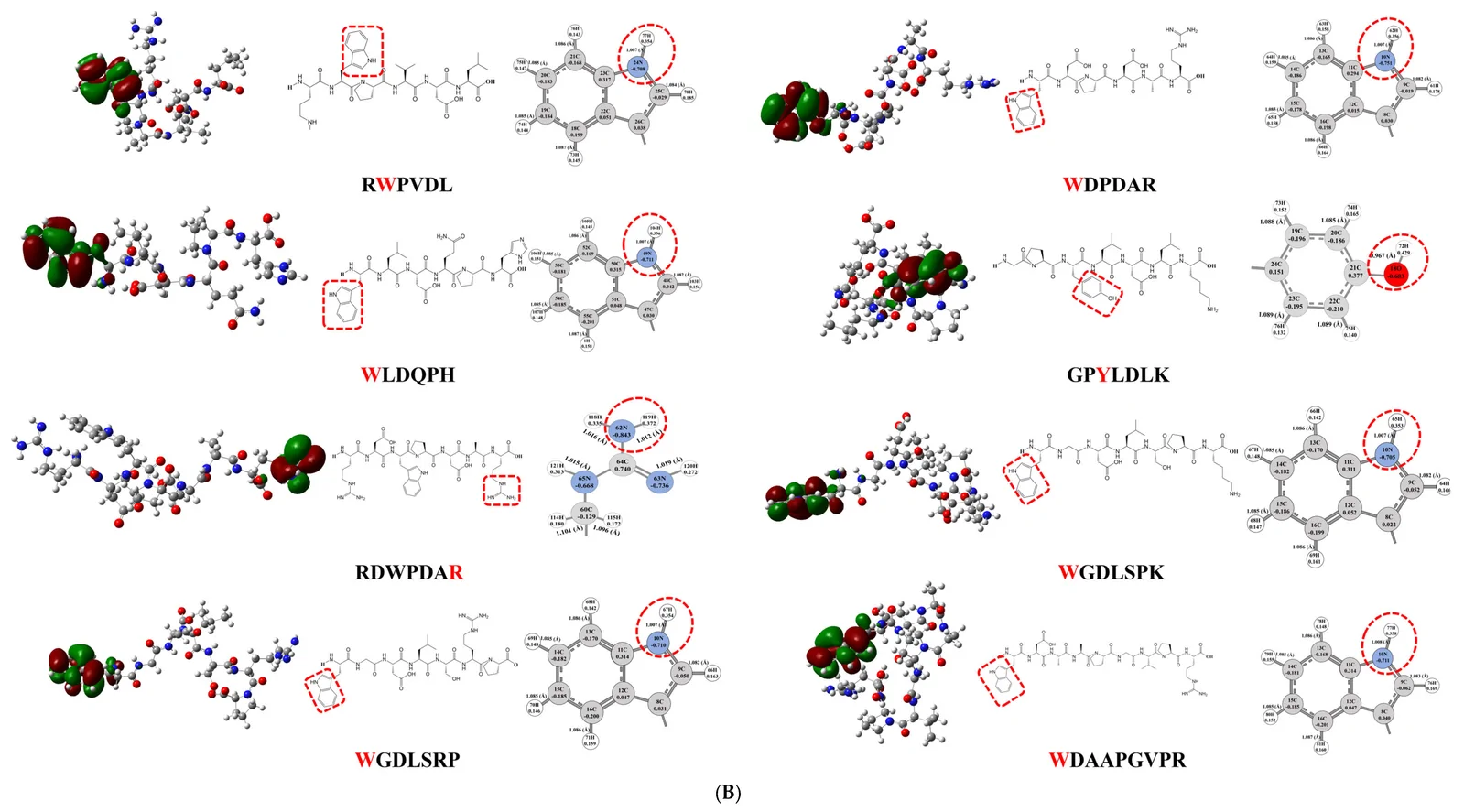
Identification of potential antioxidant-active sites in ASEB peptides based on HOMO distribution and Mulliken charge analysis: (**A**) NVGW, WALN, DFLRY, GYLPR, WGKLD, and YVDRF. (**B**) RWPVDL, WDPDAR, WLDQPH, GPYLDLK, RDWPDAR, WGDLSPK, WGDLSRP, and WDAAPGVPR. The red and green regions represent the positive and negative phases of the molecular orbital wavefunction, respectively, while the size and spatial extent of the electron density distribution indicate the distribution of electrons within the molecule.

Quantum chemical calculations were used to investigate the electronic basis of peptide antioxidant activity. Electron-donating ability is a key determinant of radical scavenging. According to frontier molecular orbital theory, the HOMO is responsible for interactions with electrophilic/radical species [43]. HOMO electron density (Figure 2) was mainly concentrated on tryptophan indole, tyrosine phenolic hydroxyl, and arginine guanidino groups, identifying them as primary reactive sites. Radical scavenging is favored in HOMO regions with high electron density and elongated bonds, which promote hydrogen transfer. Mulliken charge analysis (Table S3) further showed negative charge localization on N atoms of Trp, O atoms of Tyr, and N atoms of Arg residues, supporting their role as hydrogen donors.

Based on charge and bond length analysis, the hydrogen–donor sites responsible for radical neutralization were identified in each peptide (e.g., 32N–61H in NVGW, 10N–44H in WALN, 45O–92H in DFLRY, 40N–83H in GYLPR, 10N–52H in WGKLD, 14O–61H in YVDRF, 24N–77H in RWPVDL, 10N–62H in WDPDAR, 49N–104H in WLDQPH, 18O–72H in GPYLDLK, 63N–121H in RDWPDAR, 10N–65H in WGDLSPK, 10N–67H in WGDLSRP, and 10N–77H in WDAAPGVPR; see Table 2). Indole N–H (Trp) and hydroxyl groups (Tyr) may serve as important functional moieties involved in radical scavenging. These residues may contribute to antioxidant activity regardless of the location at the termini or within the internal sequence of the peptide chain, which is consistent with previous reports on corn-derived and ASEB peptides [26,40]. In the absence of N-terminal tryptophan, the C-terminal arginine residue may potentially participate in electron transfer as a weak electron-donating center, thereby contributing to the overall antioxidant capacity.

### 3.4. Validation of HOMO-Derived Reactive Sites

To verify the predicted HOMO-derived active sites, site-specific methylation was performed at the corresponding hydrogen atoms to block their potential antioxidant function. Specifically, the predicted active hydrogen donor sites (32N–61H, 10N–44H, 45O–92H, 40N–83H, 10N–52H, 14O–61H, 24N–77H, 10N–62H, 49N–104H, 18O–72H, 63N–121H, 10N–65H, 10N–67H, and 10N–77H) were selectively methylated. Consequently, fourteen modified peptides were synthesized, with sequences including NVGW(me), W(me)ALN, DFLRY(me), GYLPR(me), W(me)GKLD, Y(me)VDRF, RW(me)PVDL, W(me)DPDAR, W(me)LDQPH, GPY(me)LDLK, RDWPDAR(me), W(me)GDLSPK, W(me)GDLSRP, and W(me)DAAPGVPR (Table 1). In vitro antioxidant capacities of methylated peptides were then tested. A significant reduction in ABTS radical scavenging activity was observed, with values ranging from 0.00 ± 0.00 to 0.39 ± 0.00 μmol TE/μmol. These values correspond to only 0.04–5.57% of the activities of their unmodified counterparts. Among them, GYLPR(me) and W(me)DPDAR retained relatively higher activities at 18.02% and 23.02%, respectively. Similarly, significant decreases in ORAC values were also recorded, representing only 2.67–14.36% of those prior to methylation. These results validate that hydrogen atoms at the predicted active sites act as critical hydrogen donors during free radical scavenging, and that their chemical modification substantially impairs antioxidant performance. Overall, these findings further support the accuracy of HOMO–based predictions in elucidating structure–activity relationships. Consistent with these findings, previous studies have emphasized the crucial function of the indole N-H of tryptophan in mediating the antioxidant activity of peptides [26].

### 3.5. Molecular Docking for Mechanistic Validation of Antioxidant Peptides

**Figure 3:**
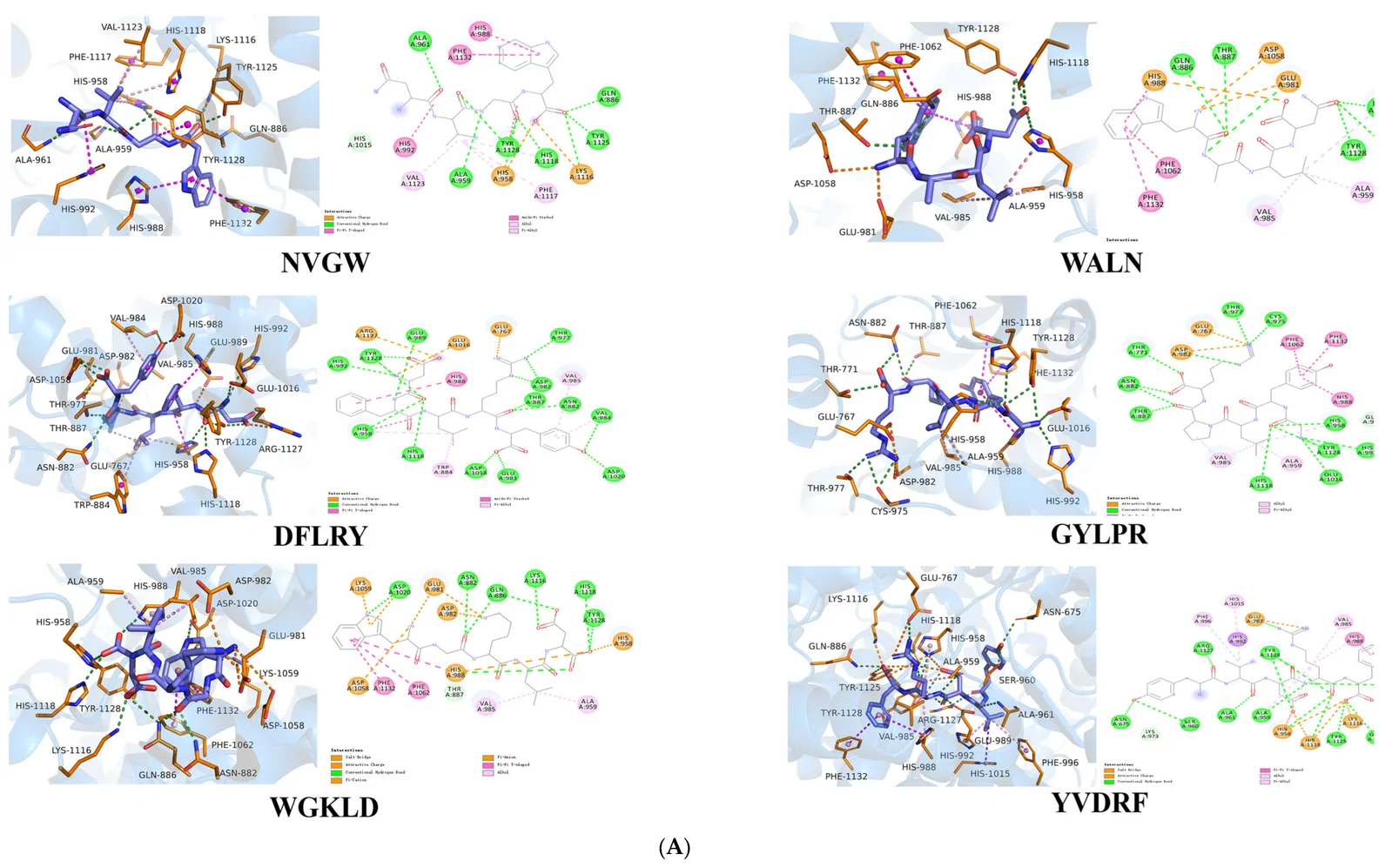

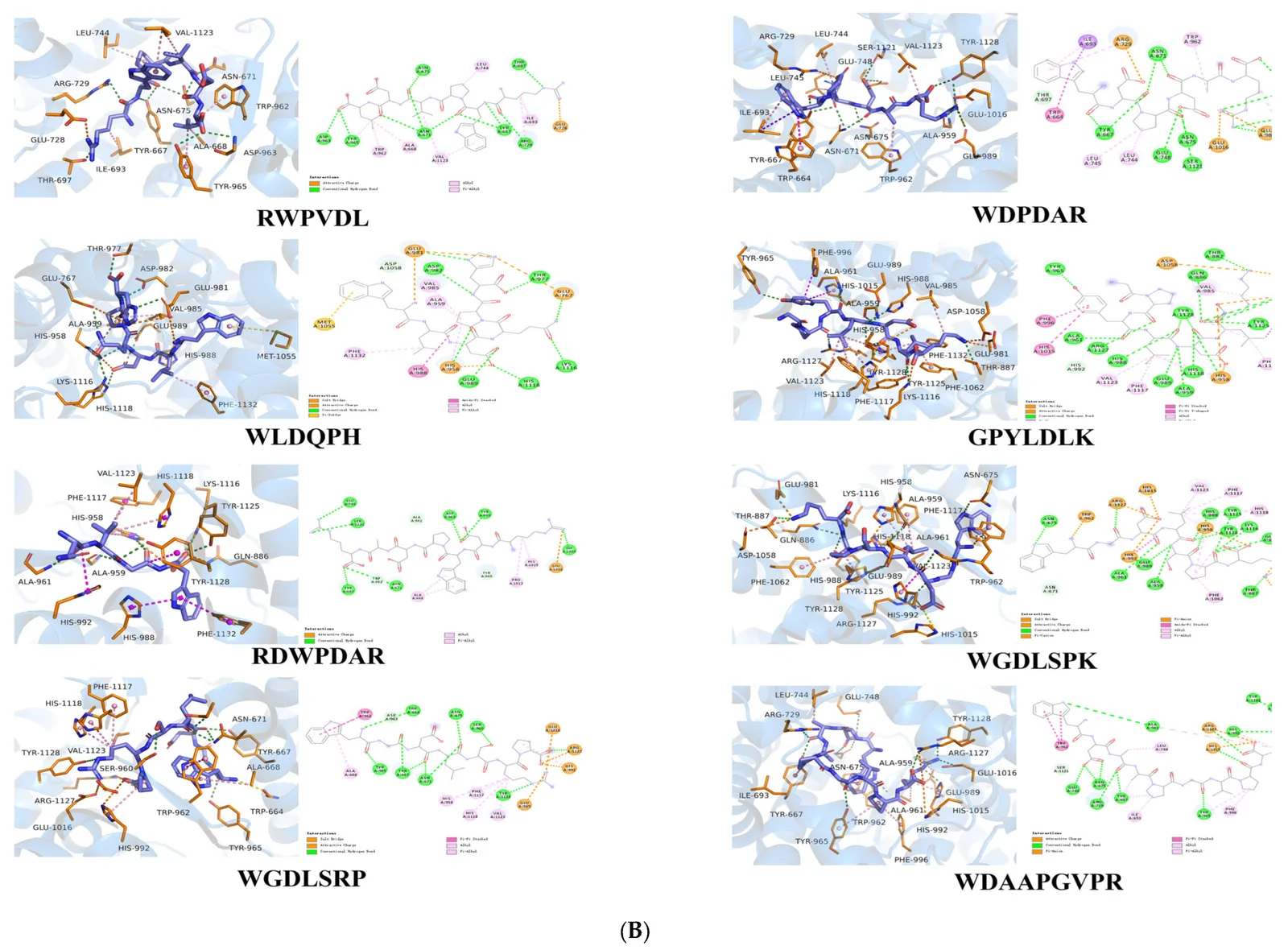
Binding conformations of fourteen ASEB peptides with MPO in 3D and 2D representations: (**A**) NVGW, WALN, DFLRY, GYLPR, WGKLD, and YVDRF. (**B**) RWPVDL, WDPDAR, WLDQPH, GPYLDLK, RDWPDAR, WGDLSPK, WGDLSRP, and WDAAPGVPR. Green is hydrogen bond; Red is electrostatic interaction; Purple is hydrophobic interaction.

As shown in Figure 3, favorable binding conformations were obtained for all fourteen peptides. The main intermolecular interactions included hydrogen bonding (conventional and carbon–hydrogen bonds), electrostatic forces (salt bridges, charge attractions, π−cation/anion interactions), and hydrophobic interactions (alkyl, π–σ, π–π, T–shaped, stacking, and π–sulfur). Binding energies < −5.0 kcal/mol and <−7.0 kcal/mol reflect moderate and strong affinity, respectively [44]. In this study, all peptides showed strong MPO binding (−8.3 to −10.6 kcal/mol) (Table S4). Three-dimensional structural visualization revealed that peptides predominantly docked into the MPO active–site cavity (Figure 3). A total of 33 MPO residues participated in interactions (Table S4). Stabilization was predominantly achieved through hydrogen bonding and electrostatic forces with residues such as Arg239, Arg333, Arg424, Asp94, Asp98, Glu102, His95, His336 and Thr100. Hydrogen bonds were chiefly localized to Arg239, Arg333, Arg424, Asp94, Asp98, Glu102, His336, and Thr100. Electrostatic interactions were mainly observed between basic residues of MPO (e.g., Arg239, Arg333, Glu102, and His336) and aromatic residues (particularly tryptophan) or C–terminal carboxyl groups of the peptides. Previous studies support these findings. Figueiredo-Rinhel et al. [45] reported that antioxidants reduce reactive oxygen species in human neutrophils by specifically interacting with His-95, Arg-239, and Gln-91 residues in the MPO catalytic site. Cai et al. [33] demonstrated that the tuna antioxidant peptide ACGSDGK forms strong hydrogen bonds, electrostatic interactions, and salt bridges with MPO residues Arg239, Arg333, Arg424, Asp94, Asp98, Glu102, and His336, thereby inhibiting MPO activity and potentially mitigating oxidative stress and inflammation in vivo.

Molecular docking analysis revealed a strong binding affinity between the ASEB-derived peptides and MPO, indicating a potential molecular interaction.

### 3.6. Investigation of the Mechanism

#### Effects on Oxidative Stress Markers in Zebrafish Embryos

**Figure 4:**
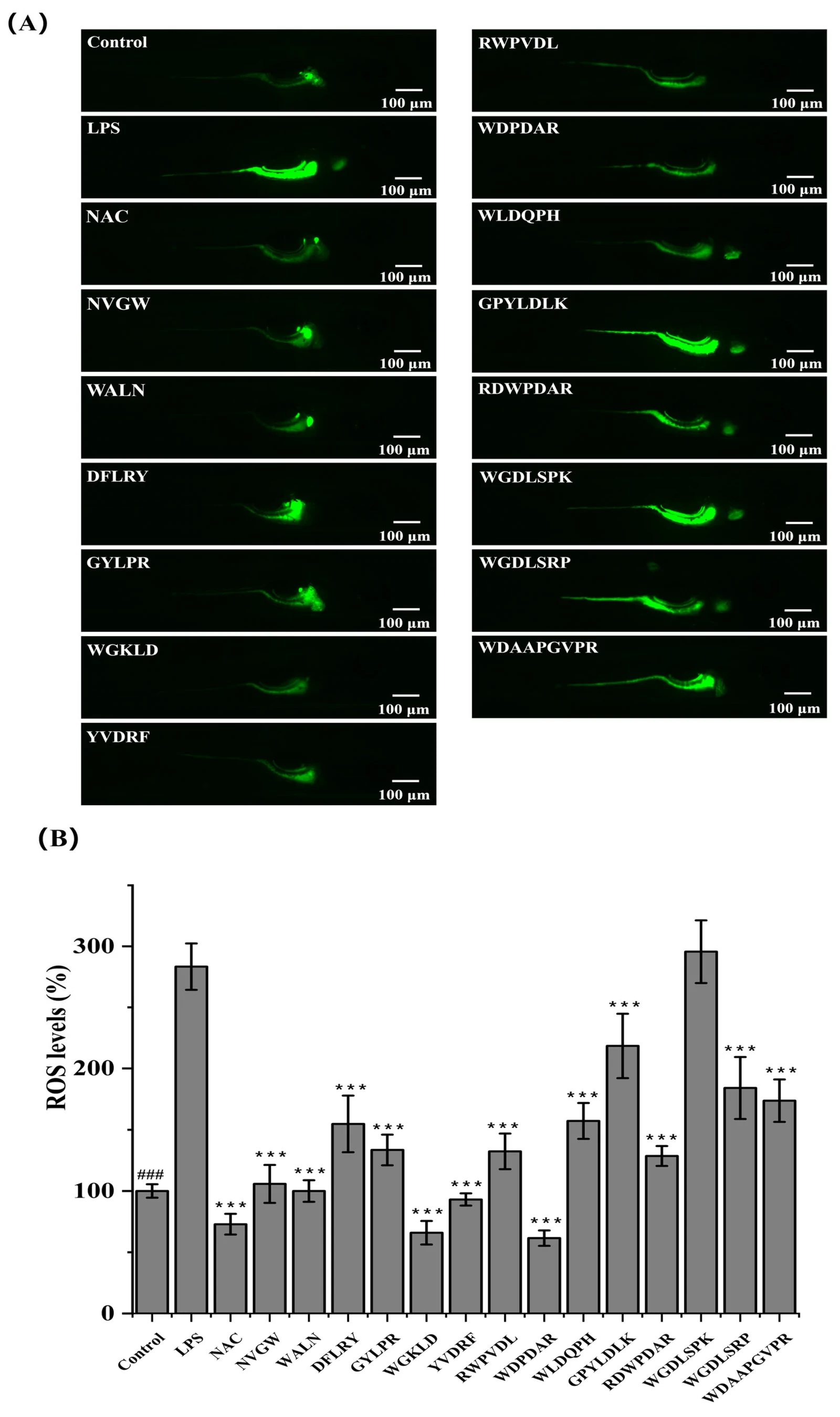
Effects of antioxidant peptides on ROS levels in zebrafish embryos: (**A**) Fluorescence images. (**B**) Quantification of fluorescence intensity using ImageJ software. **^###^** *p* < 0.001, LPS group vs. Control group; *** *p* < 0.001, peptide-treated groups vs. LPS group.

**Figure 5:**
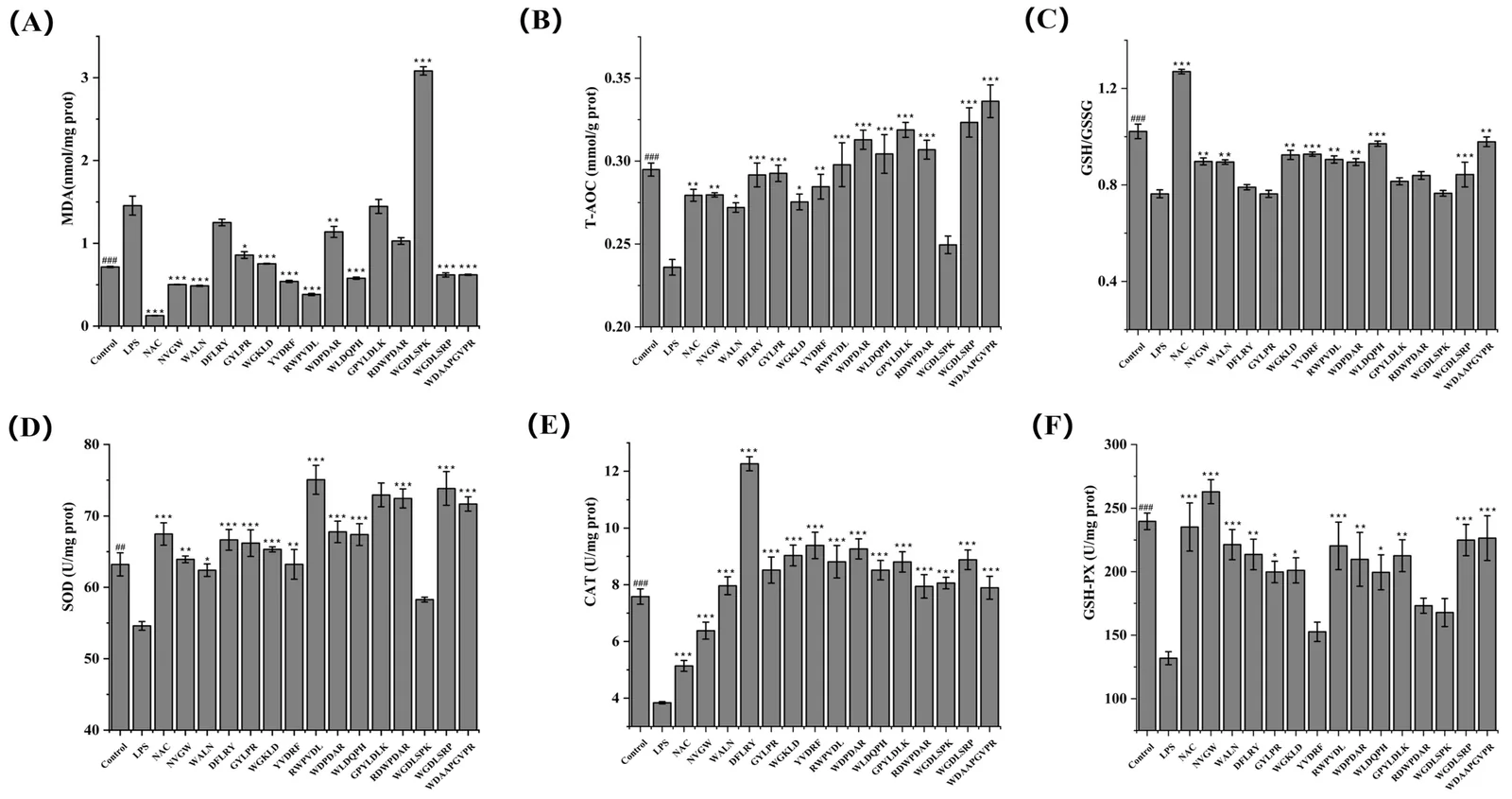
Effects of antioxidant peptides on various indicators in zebrafish embryos: (**A**) MDA, (**B**) GSH/GSSG, (**C**) SOD, (**D**) CAT, (**E**) GSH-Px, (**F**) T-AOC. ## *p* < 0.01, ### *p* < 0.001, LPS group vs. Control group; * *p* < 0.05, ** *p* < 0.01 and *** *p* < 0.001, peptide-treated groups vs. LPS group.

The DCFH-DA fluorescent probe was used to detect ROS in zebrafish embryos, generating DCF with high fluorescence intensity [35]. The green fluorescence intensity was positively correlated with intracellular ROS levels. Quantitative analysis of fluorescence microscopy images was performed using ImageJ software (version 1.54, National Institutes of Health, Bethesda, MD, USA) (Figure 4A), and the results are presented in Figure 4B. Fluorescence intensity was significantly increased following LPS treatment. In all antioxidant peptide-treated groups except WGDLSPK, fluorescence intensity was significantly lower than the model group (*p* < 0.001). Fluorescence quantification indicated that ROS levels in the model group were 2.83 ± 0.02 times those of the control group, confirming that LPS induction significantly increased ROS accumulation in zebrafish embryonic tissues (*p* < 0.001). In antioxidant peptide-treated groups, fluorescence intensity decreased by 27.56% ± 2.94% to 78.29% ± 0.99% relative to the model group. Notably, the NAC group showed a reduction of 74.29% ± 1.34%, while the WDPDAR group exhibited ROS scavenging activity exceeding that of the positive control. During oxidative stress, ROS-induced lipid peroxidation generates MDA, a biomarker of cellular damage in zebrafish [24]. MDA levels were markedly elevated in the WGDLSPK-treated group. The MDA level was measured as 3.08 ± 0.05 nmol/mg protein (Figure 5A), which was consistent with increased ROS accumulation. The next highest MDA content was observed in the GPYLDLK-treated group, with a value of 1.45 ± 0.08 nmol/mg protein. In contrast, MDA levels in the remaining peptide-treated groups were significantly lower than those in the LPS-treated group. The results indicate that oxidative stress was induced by LPS exposure in zebrafish embryos, resulting in substantial ROS accumulation. Cellular damage was effectively mitigated by treatment with several antioxidant peptides, demonstrating protective effects in vivo.

GSH serves as a key antioxidant defense [26]. A balanced GSH/GSSG ratio was observed in the control group (1.02 ± 0.03; Figure 5B), whereas LPS markedly decreased it to 0.76 ± 0.02. Peptide treatment significantly restored the ratio compared with the LPS group (*p* < 0.001). This indicated that oxidative damage was effectively alleviated through restoration of redox homeostasis. Among these groups, the highest GSH/GSSG ratios were observed in the WLDQPH and WDAAPGVPR treatments, reaching 0.97 ± 0.01 and 0.98 ± 0.02, respectively. Higher ratios were observed in these groups compared with other peptide treatments, with WGKLD and YVDRF both above 0.92 ± 0.02. Zebrafish antioxidant defense includes enzymatic systems such as SOD, CAT, and GSH-Px, essential for resisting oxidative stress. LPS exposure markedly reduced their activities (*p* < 0.001; Figure 5C–E), to 0.86-, 0.51-, and 0.55-fold of control levels. These findings indicated that LPS exposure induced excessive oxidative stress, leading to accelerated depletion of antioxidant enzymes. Following peptide treatment, enzymatic antioxidant activities were restored to varying extents compared with the LPS group. Notably, the WGDLSRP markedly increased SOD, CAT, and GSH-Px activities versus LPS (73.85, 8.88, and 224.93 U/mg protein, respectively). Significant increases in antioxidant enzyme activities were also observed in the RWPVDL, WDPDAR, WLDQPH, and GPYLDLK treatment groups (*p* < 0.001). Overall, antioxidant peptides significantly enhanced enzymatic and non-enzymatic antioxidant systems in zebrafish relative to LPS treatment, resulting in increased T-AOC. Highly significant differences were observed in thirteen peptide-treated groups (*p* < 0.001).

### 3.7. Effects of Antioxidant Peptides on Genes Related to the Keap1-Nrf2 Pathway

**Figure 6:**
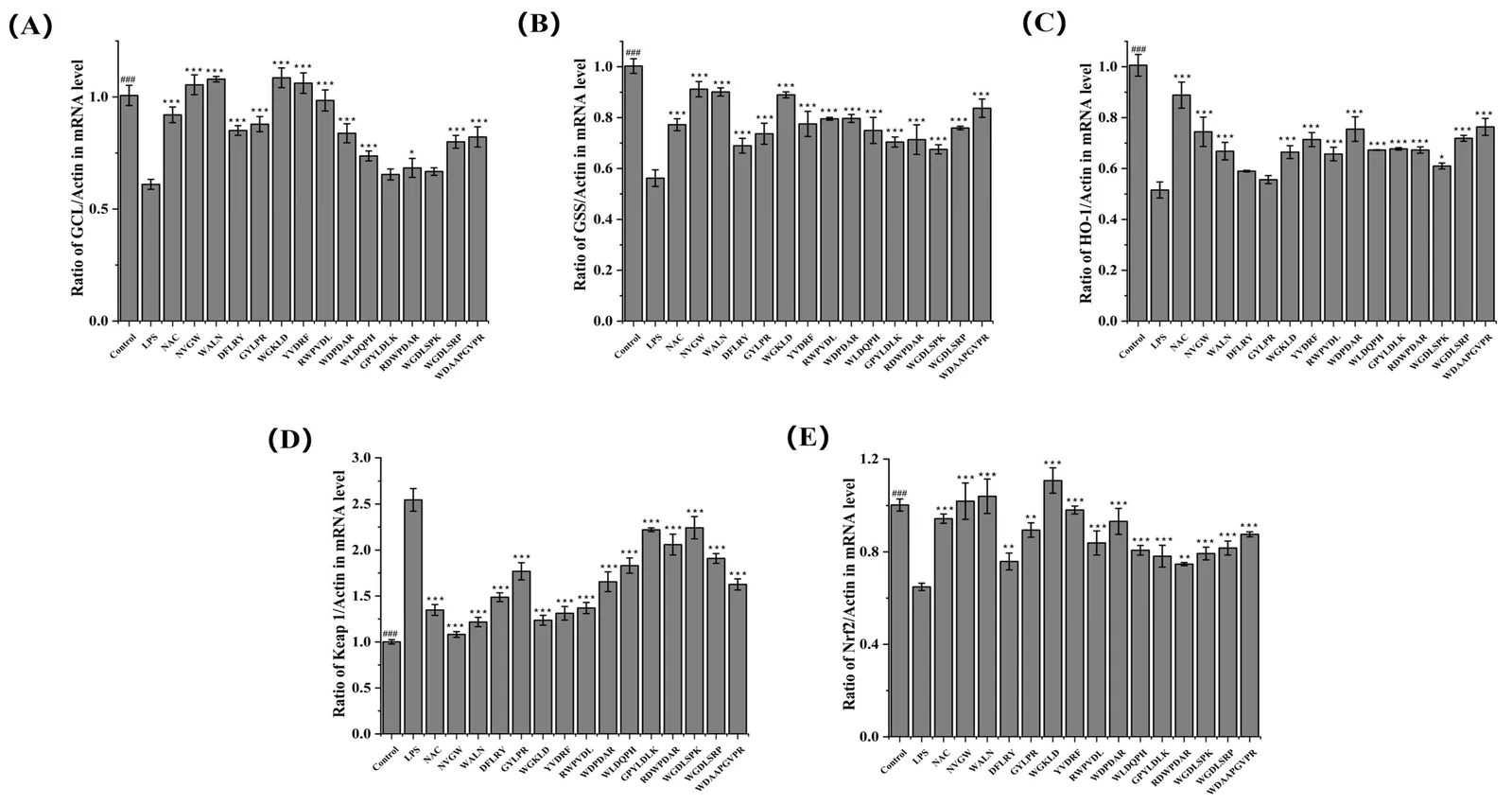
Effects of antioxidant peptides on the relative mRNA expression levels of antioxidant-related genes in zebrafish embryos: (**A**) Keap1, (**B**) Nrf2, (**C**) HO-1, (**D**) GCL and (**E**) GSS. ### *p* < 0.001, LPS group vs. Control group; * *p* < 0.05, ** *p* < 0.01 and *** *p* < 0.001, peptide-treated groups vs. LPS group.

The Keap1-Nrf2 pathway regulates endogenous antioxidant responses. Upon LPS-induced oxidative stress in zebrafish, a significant increase in Keap1 gene transcription was observed following LPS treatment (*p* < 0.001). The transcription level reached approximately 2.54-fold of that in the control group. In contrast, Nrf2 gene transcription was markedly decreased following LPS treatment (*p* < 0.001), reaching 0.65-fold of the control value. Relative to the LPS-treated group, Keap1 gene transcription was significantly downregulated following antioxidant peptide treatment. The transcription levels were reduced to 0.43–0.88 times those in the LPS-treated group. Meanwhile, Nrf2 gene transcription was significantly upregulated. The transcription levels increased to 1.15–1.71 times those in the LPS-treated group. Treatment with antioxidant peptides significantly reversed the oxidative stress-induced downregulation of antioxidant genes, including GCL, GSS, and HO-1. Specifically, LPS treatment reduced the transcription levels of GCL, GSS, and HO-1 genes to 0.61-, 0.56-, and 0.51-fold of control levels, respectively. In contrast, peptide treatments enhanced the transcription of all three genes to varying extents (Figure 6A–E). Consistent results have been reported previously by Ji et al. [46] and Wasik et al. [47]. Pearson correlation analysis showed significant correlations (*p* < 0.01) between ROS levels in zebrafish and the GSH/GSSG ratio, GSH-Px activity, and the transcription levels of GCL, GSS, HO-1, Keap1, and Nrf2 genes (Table S5). These results suggest that the antioxidant effects of ASEB peptides may be attributed to the regulation of Keap1–Nrf2 pathway-related gene expression, promotion of GSH-Px activity, and maintenance of GSH/GSSG homeostasis, which collectively contribute to the attenuation of oxidative stress.

### 3.8. Mechanistic Study of Antioxidant Peptides Inhibiting LPS-Induced Oxidative Damage in Zebrafish

**Figure 7:**
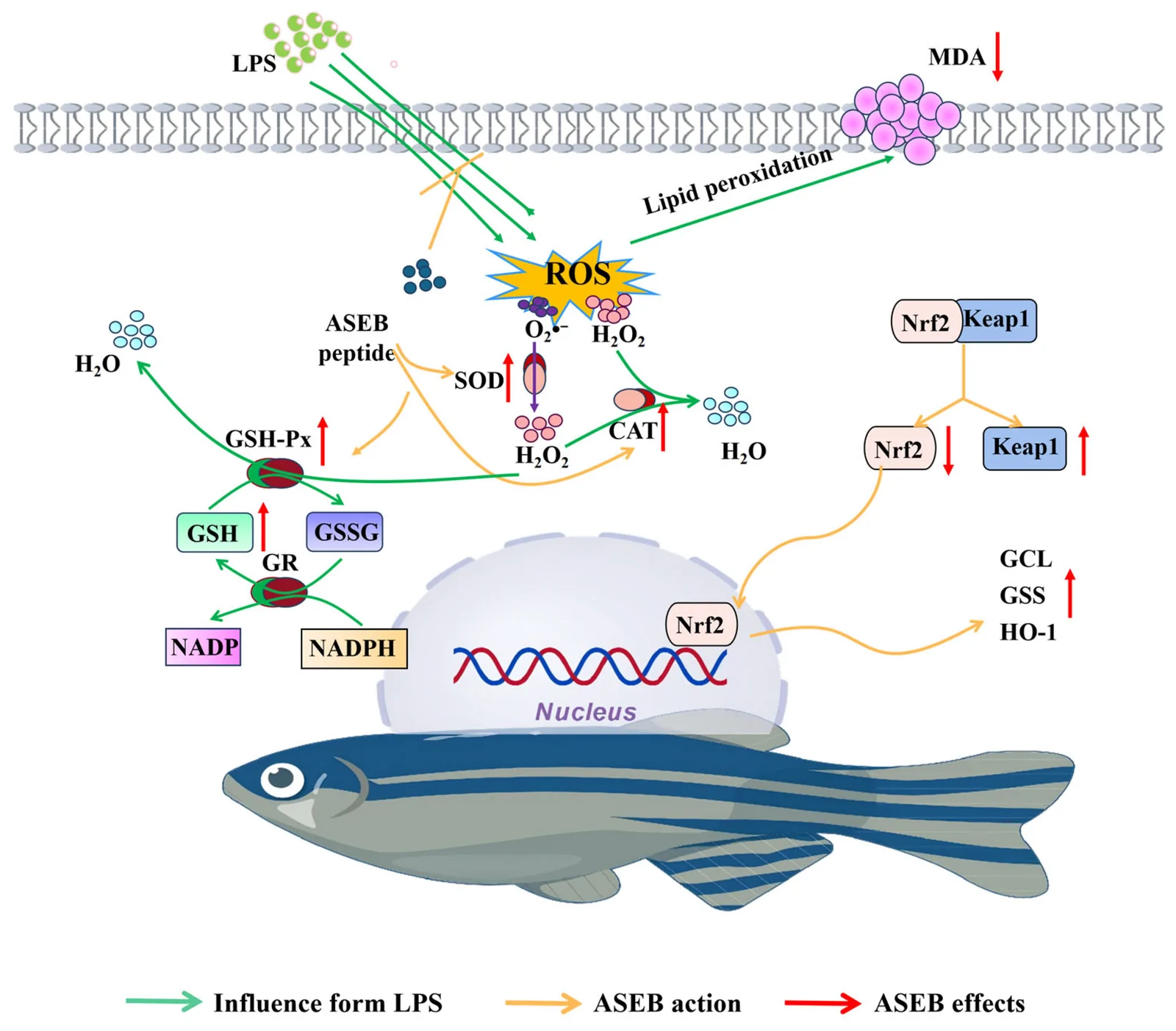
Potential mechanism of antioxidant peptides in mitigating LPS-induced oxidative damage in zebrafish embryos. Green arrows indicate the influence of LPS, orange arrows indicate ASEB action, and red arrows indicate the effects of ASEB.

As shown in Figure 7, LPS is recognized as a free radical initiator that induces substantial production of ROS upon exposure in zebrafish [48,49,50]. ROS overproduction triggers lipid peroxidation in cell membranes, resulting in elevated MDA levels. Free radical levels in zebrafish are reduced by antioxidant peptides through direct interaction with peroxy radicals generated by LPS. GSH, a primary non-enzymatic antioxidant defense in zebrafish. Through GSH-Px activity, GSH is converted to GSSG, which is recycled to GSH via GR and NADPH, thereby limiting lipid peroxidation. In addition to non-enzymatic antioxidants, an enzymatic antioxidant defense system is present in zebrafish, including SOD, CAT, and GSH-Px. SOD transforms O_2_^−^ into H_2_O_2_, followed by CAT-mediated decomposition into water. Meanwhile, H_2_O_2_ is further detoxified by GSH-Px through conversion to water or reaction with GSH. Both antioxidant systems cooperatively regulate redox homeostasis by balancing ROS formation and removal. Redox homeostasis is thereby sustained through direct or indirect participation in cellular redox reactions, supporting normal physiological functions. However, prolonged exposure to excessive free radicals overwhelms the intracellular defense system. As a consequence, effective elimination of radicals is impeded, resulting in oxidative damage. ASEB-derived antioxidant peptides enhanced the antioxidant capacity of zebrafish and alleviated LPS-induced oxidative damage, which was associated with the regulation of Keap1—Nrf2-related antioxidant gene expression, upregulation of HO-1 and glutathione synthesis-related enzymes (GCL and GSS), restoration of the GSH/GSSG ratio, and activation of antioxidant enzymes, including SOD, CAT, and GSH-Px. Overall, ASEB-derived antioxidant peptides may help attenuate oxidative damage in zebrafish.

## 4. Conclusions

In this study, an integrated strategy combining antioxidant activity evaluation and in silico screening was employed to identify 14 novel antioxidant peptides (NVGW, WALN, DFLRY, GYLPR, WGKLD, YVDRF, RWPVDL, WDPDAR, WLDQPH, GPYLDLK, RDWPDAR, WGDLSPK, WGDLSRP, and WDAAPGVPR), all of which exhibited stronger free radical scavenging activities than the positive control. Quantum chemical analysis revealed that the hydrogen-donating groups within tryptophan indole, tyrosine phenolic hydroxyl, and arginine guanidinium moieties played critical roles in determining the antioxidant potential of these peptides. Moreover, the zebrafish embryo oxidative stress model demonstrated that ASEB-derived antioxidant peptides effectively attenuated oxidative stress by modulating Keap1—Nrf2-related antioxidant gene expression and enhancing the endogenous antioxidant defense system. Collectively, the multidimensional screening approach established in this study provides a promising strategy for the efficient discovery of novel antioxidant peptides, while supporting the high-value conversion of ASEB byproducts into functional food ingredients. Further investigations integrating mammalian cell models, real-time ROS monitoring, peptide absorption and transport evaluation, Western blotting, and immunofluorescence analyses are warranted to elucidate the precise molecular targets and underlying mechanisms of these bioactive peptides.

## Figures and Tables

**Table 1:** Physicochemical properties and antioxidant activities of the fourteen peptides and their modified peptides from ASEB. Data with different letters in the same test indicate significant differences (*p* < 0.05).

Peptide Sequence	Marks	Hydrophobicity	Steric Hindrance	Amphipathy	ABTS Assay (μmol TE/μmol)	ORAC Assay (μmol TE/μmol)	Modified Peptides		
							Peptide Sequence	ABTS Assay (μmol TE/μmol)	ORAC Assay (μmol TE/μmol)
NVGW	0.63	0.11	0.66	0	2.76 ± 0.04 ^e^	4.00 ± 0.07 ^e^	NVGW(me)	0.15 ± 0.00 ^d^	0.31 ± 0.00 ^i^
WALN	0.71	0.13	0.58	0	3.02 ± 0.03 ^d^	4.63 ± 0.21 ^cd^	W(me)ALN	0.12 ± 0.00 ^fg^	0.33 ± 0.00 ^h^
DFLRY	0.75	–0.26	0.67	0.49	2.33 ± 0.02 ^f^	3.30 ± 0.20 ^g^	DFLRY(me)	0.00 ± 0.00 ^ij^	0.10 ± 0.00 ^jk^
GYLPR	0.71	–0.22	0.59	0.49	1.89 ± 0.05 ^h^	3.60 ± 0.14 ^f^	GYLPR(me)	0.34 ± 0.00 ^b^	0.40 ± 0.01 ^g^
WGKLD	0.60	–0.15	0.63	0.73	2.96 ± 0.11 ^d^	4.88 ± 0.10 ^c^	W(me)GKLD	0.14 ± 0.01 ^e^	0.41 ± 0.00 ^f^
YVDRF	0.63	–0.26	0.71	0.49	4.30 ± 0.12 ^a^	2.79 ± 0.14 ^h^	Y(me)VDRF	0.00 ± 0.00 ^j^	0.09 ± 0.00 ^k^
RWPVDL	0.71	–0.19	0.59	0.41	2.38 ± 0.03 ^f^	3.58 ± 0.17 ^fg^	RW(me)PVDL	0.07 ± 0.00 ^h^	0.51 ± 0.00 ^e^
WDPDAR	0.76	–0.44	0.6	0.41	1.70 ± 0.02 ^i^	4.60 ± 0.18 ^cd^	W(me)DPDAR	0.39 ± 0.00 ^a^	0.51 ± 0.00 ^e^
WLDQPH	0.61	–0.16	0.47	0.45	2.14 ± 0.03 ^g^	5.26 ± 0.17 ^b^	W(me)LDQPH	0.07 ± 0.00 ^h^	0.51 ± 0.01 ^e^
GPYLDLK	0.62	–0.09	0.61	0.52	3.22 ± 0.14 ^c^	4.11 ± 0.11 ^e^	GPY(me)LDLK	0.01 ± 0.00 ^i^	0.11 ± 0.01 ^j^
RDWPDAR	0.69	–0.63	0.61	0.7	3.65 ± 0.02 ^b^	4.50 ± 0.10 ^d^	RDWPDAR(me)	0.12± 0.00 ^f^	0.61 ± 0.01 ^b^
WGDLSPK	0.63	–0.16	0.58	0.52	2.67 ± 0.11 ^e^	4.90 ± 0.06 ^c^	W(me)GDLSPK	0.11 ± 0.01 ^g^	0.53 ± 0.00 ^d^
WGDLSRP	0.71	–0.25	0.58	0.35	3.21 ± 0.04 ^c^	4.86 ± 0.25 ^c^	W(me)GDLSRP	0.18 ± 0.01 ^c^	0.55 ± 0.00 ^c^
WDAAPGVPR	0.75	–0.12	0.56	0.27	3.29 ± 0.06 ^c^	5.90 ± 0.28 ^a^	W(me)DAAPGVPR	0.15 ± 0.00 ^de^	0.65 ± 0.00 ^a^

**Table 2:** Mulliken charge distribution and bond lengths at predicted antioxidant active sites in fourteen ASEB-derived peptides.

Peptide	Mulliken Charge		Bond Length (Å)		Active Site
NVGW	32N	–0.687	32N–61H	1.011	32N–61H
	61H	0.354			
WALN	10N	–0.706	10N–44H	1.008	10N–44H
	44H	0.354			
DFLRY	45O	–0.680	45O–92H	0.978	45O–92H
	92H	0.429			
GYLPR	40N	–0.846	40N–83H	1.013	40N–83H
	83H	0.370			
WGKLD	10N	–0.709	10N–52H	1.007	10N–52H
	52H	0.353			
YVDRF	14O	–0.681	14O–61H	0.967	14O–61H
	61H	0.428			
RWPVDL	24N	–0.708	24N–77H	1.007	24N–77H
	77H	0.354			
WDPDAR	10N	–0.751	10N–62H	1.007	10N–62H
	62H	0.356			
WLDQPH	49N	–0.711	49N–104H	1.007	49N–104H
	104H	0.356			
GPYLDLK	18O	–0.683	18O–72H	0.967	18O–72H
	72H	0.426			
RDWPDAR	63N	–0.736	63N–121H	1.019	63N–121H
	121H	0.313			
WGDLSPK	10N	–0.705	10N–65H	1.007	10N–65H
	65H	0.353			
WGDLSRP	10N	–0.710	10N–67H	1.007	10N–67H
	67H	0.354			
WDAAPGVPR	10N	–0.711	10N–77H	1.008	10N–77H
	77H	0.358			

## Data Availability

The original data presented in this study are openly available through the following public repositories and online resources. Peptide bioactivity prediction was performed using PeptideRanker (http://distilldeep.ucd.ie/PeptideRanker/, accessed on 26 August 2022). Allergenicity and toxicity predictions were conducted using AllerTOP v.2.0 (https://ddg-pharmfac.net/AllerTOP/, accessed on 2 September 2022) and ToxinPred (https://webs.iiitd.edu.in/raghava/toxinpred/, accessed on 6 September 2022), respectively. To assess the novelty of the identified peptides, an exact sequence matching approach was performed against the BIOPEP-UWM database and the NCBI Protein database (accessed 15 October 2022). No peptides with identical sequences and previously reported antioxidant activities were identified in either database. The reference gene sequences used for primer design and qPCR analysis were retrieved from the NCBI GenBank/RefSeq database (accessed 12 December 2025), including Keap1 (NM_182864.2), Nrf2 (NM_182889.1), HO-1 (NM_001127516.1), GCL (NM_207050.1), GSS (NM_001006104.1), and β-actin (NM_131031.2). No sequencing data were generated in the present study.

## Supplementary Materials

The following supporting information can be downloaded at https://www.mdpi.com/article/10.3390/antiox15091057/s1, Table S1: Primers sequence used for RT-qPCR analysis in zebrafish; Table S2: In silico screening of peptides, including their predicted physicochemical properties and activity scores; Table S3: Atomic charge distribution of predicted active sites of the fourteen peptides; Table S4: Docking Sites and Interaction Forces Between Synthetic Peptides and MPO Receptors; Table S5: Pearson’s correlation coefficients between ROS and other components (** represent a highly significant); Figure S1: Antioxidant activity (A) and protein recovery, degree of proteolysis (B) of ASEB enzymolysis products; Figure S2: Chromatogram of the alcalase hydrolysate of ASEB on Sephadex G–25 (A) and antioxidant activity of each component (B); Figure S3: Identification of antioxidant peptides NVGW and WALN from F4 fraction utilizing LC–MS/MS; Figure S4: Effects of antioxidant peptides on mortality (A–C) and deformation (D–F) in zebrafish embryos.

## Abbreviations

The following abbreviations are used in this manuscript:

ASEB	Asian swamp eel bone
MPO	Myeloperoxidase
BV/TV	Bone volume/total tissue volume
Trolox	6-Hydroxy-2,5,7,8-tetramethylxanthen-2-carboxylic acid
AAPH	2,2′-azobis [2-methylpropionamide] dihydrochloride
ABTS	2,2′-Azino-bis(3-ethylbenzothiazol-5-sulfonic acid)
DCFH-DA	2′,7′-Dichlorofluorescin diacetate
NAC	N-acetyl-L-cysteine
BCA	Bicinchoninic acid
CAT	Catalase
MDA	Malondialdehyde
GSH-Px	Glutathione peroxidase
SOD	Superoxide dismutase
GSH	Reduced glutathione
GSSG	Oxidized glutathione
T-AOC	Total antioxidant capacity
DH	Degree of hydrolysis
OPA	O-phthalaldehyde
SDS	Sodium dodecyl sulfate
DTT	Dithiothreitol
TEAC	Trolox equivalent antioxidant capacity
ORAC	Oxygen radical absorption capacity
DFT	Density functional theory
NZRC	National Zebrafish Resource Center
G	Glycine
P	Proline
L	Leucine
A	Alanine
R	Arginine
D	Aspartic acid
Y	Tyrosine
W	Tryptophan
K	Lysine
N	Asparagine
