# Supplementary material for: Identification and In Silico Selection of Novel Antioxidant Peptides from Asian Swamp Eel Bone: Quantum Chemical Calculations, Molecular Docking, and Zebrafish Model Validation
Source: Antioxidants (Basel). 2026 Aug 24;15(9):1057. doi: 10.3390/antiox15091057 (PMC13603264; doi:10.3390/antiox15091057)
Supplement: Supplementary file 1 [file antioxidants-15-01057-s001.zip › antioxidants-4452780-supplementary.pdf]

Table S1 Primers sequence used for RT-qPCR analysis in zebrafish

Primer name	Forward primer sequence	Reverse primer sequence	Size（bq）	Accession number
Keap l	CCGCCTCCAGTCCTTACTTC	AGGTCTCGCCAACTGTGATG	150	NM_182864.2
Nrf2	ACTCCAAACCTCCGTTCACC	AGTCGTCTACGGGCAGATTG	135	NM_182889.1
HO-1	AAATCACTGGGGCTGACTGG	GTGCTTCCTGCCTCTTTTGC	139	NM_001127516.1
GCL	CCATCAACCGACACCCAAGA	TGAATGGAGACGGGGTGTTG	117	NM_207050.1
GSS	TCAGAGGGAGGGAGGAGGTA	GATGTTCTGGACTGGGACGG	133	NM_001006104.1
β-Actin	AGGAGAAGCTGTGCTACGTG	CCATACCCAGGAAGGAAGGC	171	NM_131031.2

Table S2 *In silico* screening of peptides, including their predicted physicochemical properties and activity scores

	Peptide sequence	Marks	Hydrophobicity	Steric hindrance	Amphipathy
1	ALGWDH	0.61	0.03	0.5	0.24
2	DFLRY	0.75	-0.26	0.67	0.49
3	DGRGYPGER	0.51	-0.49	0.66	0.69
4	FGAGPPCHYSGPK	0.82	-0.04	0.53	0.39
5	FYCR	0.96	-0.27	0.68	0.61
6	GPYLDLK	0.62	-0.09	0.61	0.52
7	GYAVAAPGF	0.66	0.24	0.6	0
8	GYLPR	0.71	-0.22	0.59	0.49
9	LWGKDL	0.60	-0.04	0.61	0.61
10	NGDGDHGSRKYCPGAK	0.58	-0.12	0.53	0.21
11	NGDHGPQPWWPGAK	0.88	-0.16	0.54	0.46
12	NVGW	0.63	0.11	0.66	0
13	PGWSTH	0.54	-0.06	0.43	0.24
14	RDWPDAR	0.69	-0.63	0.61	0.7
15	RGDGFGDWR	0.85	-0.39	0.68	0.54
16	RWPVDL	0.71	-0.19	0.59	0.41
17	SDPMYRP	0.82	-0.28	0.62	0.41
18	SDSPTYPR	0.58	-0.41	0.56	0.31
19	SDYQLR	0.55	-0.48	0.65	0.62
20	SGPDFRDY	0.74	-0.34	0.65	0.31
21	SYAVGF	0.73	0.22	0.64	0
22	SYPHCER	0.57	-0.44	0.51	0.74
23	TDPMYPR	0.70	-0.36	0.6	0.35
24	TGPLFY	0.80	0.18	0.58	0
25	TSGVPWAW	0.73	0.15	0.54	0
26	WALN	0.71	0.13	0.58	0
27	WDAAPGVPR	0.75	-0.12	0.56	0.27
28	WDPDAR	0.76	-0.44	0.6	0.41
29	WDTRPSL	0.55	-0.3	0.56	0.35
30	WGDLSPK	0.63	-0.16	0.58	0.52
31	WGDLSRP	0.71	-0.25	0.58	0.35
32	WGGDLSPK	0.64	-0.12	0.59	0.46
33	WGGVM	0.90	0.3	0.67	0
34	WGKLD	0.60	-0.15	0.63	0.73
35	WLDQPH	0.61	-0.16	0.47	0.45
36	WPNFKY	0.91	-0.14	0.62	0.61
37	WRFL	0.99	-0.06	0.6	0.61
38	WTGL	0.81	0.22	0.56	0

39	WYDK	0.61	-0.36	0.66	0.92
40	YADRF	0.79	-0.32	0.67	0.49
41	YFDGRG	0.76	-0.26	0.7	0.41
42	YGDFK	0.70	-0.21	0.7	0.73
43	YGVPALP	0.50	0.19	0.55	0
44	YKDFF	0.91	-0.12	0.71	0.73
45	YVDRF	0.63	-0.26	0.71	0.49

Table S3 Atomic charge distribution of predicted active sites of the fourteen peptides

Peptide	NVGW		WALN		DFLRY		GYLPR		WGKLD		YVDRF		RWPVDL		WDPDAR		WLDQPH		GPYLDLK		RDWPDAR		WGDLSPK		WGDLSRP		WDAAPGVPR	
	26C	-0.210	8C	0.022	45O	-0.680	38C	-0.131	8C	0.030	3C	-0.349	18C	-0.199	8C	0.030	47C	0.030	18O	-0.683	60C	-0.129	8C	0.022	8C	0.031	8C	0.040
	27C	-0.168	9C	-0.051	46C	-0.196	39C	-0.357	9C	-0.053	8C	0.098	19C	-0.184	9C	-0.019	48C	-0.042	19C	-0.196	61C	-0.348	9C	-0.052	9C	-0.050	9C	-0.062
	28C	-0.198	10N	-0.706	47C	-0.186	40N	-0.846	10N	-0.709	9C	-0.197	20C	-0.183	10N	-0.751	49N	-0.711	20C	-0.186	62N	-0.843	10N	-0.705	10N	-0.710	10N	-0.711
	29C	-0.201	11C	0.312	48C	0.373	41N	-0.739	11C	0.314	10C	-0.190	21C	-0.168	11C	0.294	50C	0.315	21C	0.377	63N	-0.736	11C	0.311	11C	0.314	11C	0.314
	30C	0.083	12C	0.052	49C	-0.222	42C	0.740	12C	0.047	11C	0.370	22C	0.051	12C	0.015	51C	0.048	22C	-0.210	64C	0.740	12C	0.052	12C	0.047	12C	0.047
	31C	0.293	13C	-0.168	50C	-0.199	43N	-0.674	13C	-0.170	12C	-0.228	23C	0.317	13C	-0.165	52C	-0.169	23C	-0.195	65N	-0.668	13C	-0.170	13C	-0.170	13C	-0.168
**Mulliken charge**	32N	-0.687	14C	-0.182	51C	0.144	78H	0.173	14C	-0.181	13C	-0.215	24N	-0.708	14C	-0.186	53C	-0.181	24C	0.151	114H	0.180	14C	-0.182	14C	-0.182	14C	-0.181
	33C	-0.060	15C	-0.185	52C	-0.382	79H	0.170	15C	-0.186	14O	-0.681	25C	-0.029	15C	-0.178	54C	-0.185	25C	-0.391	115H	0.172	15C	-0.186	15C	-0.185	15C	-0.185
	34C	0.105	16C	-0.200	92H	0.429	80H	0.191	16C	-0.199	53H	0.171	26C	0.038	16C	-0.198	55C	-0.201	72H	0.426	116H	0.174	16C	-0.199	16C	-0.200	16C	-0.201
	57H	0.150	43H	0.163	93H	0.159	81H	0.168	51H	0.165	54H	0.172	73H	0.145	61H	0.178	1H	0.158	73H	0.152	117H	0.177	64H	0.166	66H	0.163	76H	0.169
	58H	0.141	44H	0.354	94H	0.171	82H	0.336	52H	0.353	57H	0.158	74H	0.144	62H	0.356	103H	0.156	74H	0.165	118H	0.335	65H	0.353	67H	0.354	77H	0.358
	59H	0.148	45H	0.143	95H	0.148	83H	0.370	53H	0.141	58H	0.169	75H	0.147	63H	0.158	104H	0.356	75H	0.140	119H	0.372	66H	0.142	68H	0.142	78H	0.148
	60H	0.140	46H	0.150	96H	0.154	84H	0.272	54H	0.147	59H	0.147	76H	0.143	64H	0.159	105H	0.145	76H	0.132	120H	0.272	67H	0.148	69H	0.148	79H	0.155
	61H	0.354	47H	0.148	97H	0.189	85H	0.325	55H	0.145	60H	0.164	77H	0.354	65H	0.158	106H	0.151	77H	0.186	121H	0.313	68H	0.147	70H	0.146	80H	0.152
	62H	0.183	48H	0.160	98H	0.166	86H	0.166	56H	0.160	61H	0.428	78H	0.185	66H	0.164	107H	0.148	78H	0.167	122H	0.157	69H	0.161	71H	0.159	81H	0.160
	26C-57H	1.087	9C-43H	1.082	45O-92H	0.978	38C-78H	1.101	9C-51H	1.082	3C-53H	1.094	18C-73H	1.087	9C-61H	1.082	48C-103H	1.082	18O-72H	0.967	60C-114H	1.101	9C-64H	1.082	9C-66H	1.082	9C-76H	1.083
	27C-58H	1.086	10N-44H	1.008	46C-93H	1.088	38C-79H	1.099	10N-52H	1.007	9C-57H	1.088	19C-74H	1.085	10N-62H	1.007	49N-104H	1.007	19C-73H	1.088	60C-115H	1.096	10N-65H	1.007	10N-67H	1.007	10N-77H	1.008
**Bond length (Å)**	28C-59H	1.085	13C-45H	1.086	47C-94H	1.085	40N-82H	1.013	13C-53H	1.086	10C-58H	1.085	20C-75H	1.085	13C-63H	1.086	52C-105H	1.086	20C-74H	1.085	62N-118H	1.016	13C-66H	1.086	13C-68H	1.086	13C-78H	1.086
	29C-60H	1.085	14C-46H	1.085	49C-95H	1.086	40N-83H	1.013	14C-54H	1.085	12C-59H	1.088	21C-76H	1.086	14C-64H	1.085	53C-106H	1.085	22C-75H	1.089	62N-119H	1.012	14C-67H	1.085	14C-69H	1.085	14C-79H	1.085
	32N-61H	1.011	15C-47H	1.085	50C-96H	1.089	41N-84H	1.019	15C-55H	1.085	13C-60H	1.088	24N-77H	1.007	15C-65H	1.085	54C-107H	1.085	23C-76H	1.089	63N-120H	1.019	15C-68H	1.085	15C-70H	1.085	15C-80H	1.085
	33C-62H	1.083	16C-48H	1.087	52C-97H	1.095	43N-85H	1.016	16C-56H	1.086	14O-61H	0.967	25C-78H	1.084	16C-66H	1.086	55C-1H	1.087	25C-77H	1.097	65N-121H	1.015	16C-69H	1.086	16C-71H	1.086	16C-81H	1.087
**Active site**	32N-61H		10N-44H		45O-92H		40N-83H		10N-52H		14O-61H		24N-77H		10N-62H		49N-104H		18O-72H		63N-121H		10N-65H		10N-67H		10N-77H	

Table S4 Docking Sites and Interaction Forces Between Synthetic Peptides and MPO Receptors

Note: "+"indicates the number of forces between peptide and receptor.(Green is hydrogen bond ; Red is electrostatic interaction ; Purple is hydrophobic interaction.)

		NVGW	WALN	DFLRY	GYLPR	WGKLD	YVDRF	RWPVDL	WDPDAR	WLDQ PH	GPYL DLK	RDWP DAR	WGDL SPK	WGDL SRP	WDAAPG VPR
	CDOCKER Energy (kcal/mol)	-8.4	-9.3	-9.2	-9.2	-8.6	-9.2	-9.7	-10.6	-10.4	-8.3	-9.4	-9.1	-9.8	-9.2
	Arg94						+								
	Arg98													+	
	Arg239	+++	++	++	+	+	+	+	++	++	+	+	++	+++	+
	Arg333	++	++	++	+	+++	+	+	+	+++	+		++	++	+
	Arg424	+	+	+	+	++		+	++	+	+	+	+	++	+
	Asp94	+			+	+		+		+		++	+		+
MPO	Asp98	+			+	+	+	+			+	+			+
	Gly90				+								+		
	Gln91						+		+					+	+
	Glu90							+							
	Glu102		+++	+	+	++	+	++			+	+		++	
	Glu116												+	+	
	Glu242			+											
	His95		+	+		+			++					++	+
	His96										+				

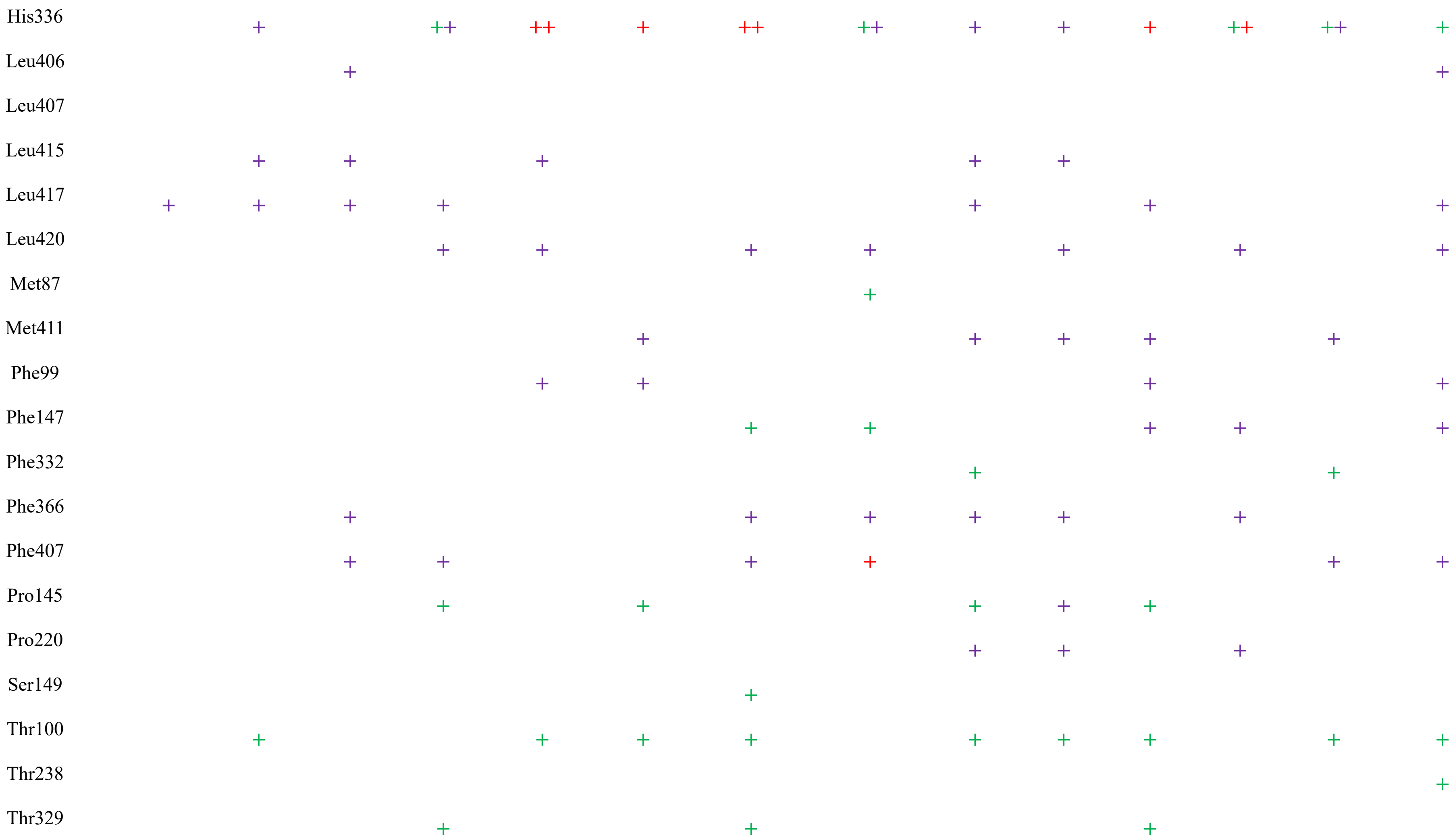
His336
Leu406
Leu407
Leu415
Leu417
Leu420
Met87
Met411
Phe99
Phe147
Phe332
Phe366
Phe407
Pro145
Pro220
Ser149
Thr100
Thr238
Thr329

Val410

+

Table S5 Pearson's correlation coefficients between ROS and other components. (** represent a highly significant

Peptide	ROS
**MDA**	**0.718****
**GSH/GSSG**	**-0.529****
**GSH-Px**	**-0.393****
**Keap 1**	**0.814****
**Nrf2**	**-0.683****
**HO-1**	**-0.469****
**GCL**	**-0.718****
**GSS**	**-0.620****

Figure S1 Antioxidant activity (A) and protein recovery, degree of proteolysis (B) of ASEB enzymolysis products

(A)

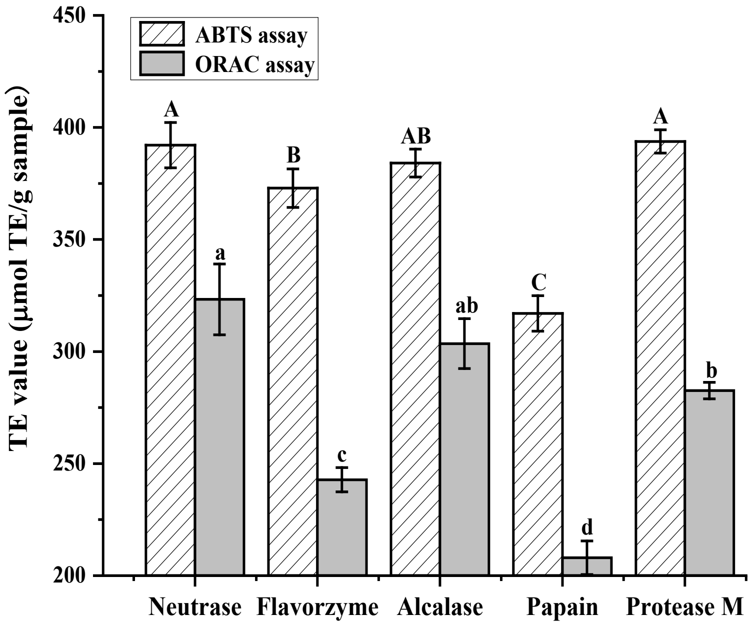

(B)

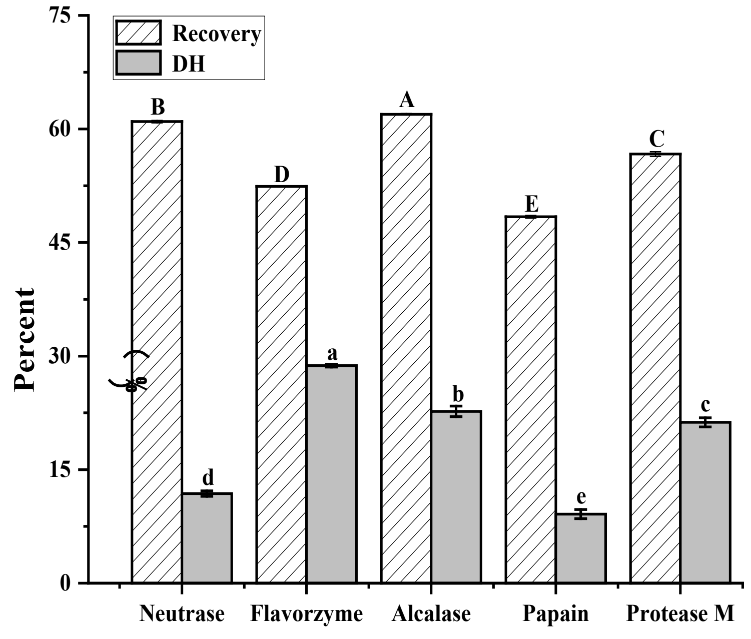

Note: Different lowercase letters (a–e) or different uppercase letters (A-D) indicate statistically significant differences among groups, as determined by one–way ANOVA followed by post hoc multiple comparisons ($p < 0.05$).

Figure S2 Chromatogram of the alcalase hydrolysate of ASEB on Sephadex G–25 (A) and antioxidant activity of each component (B)

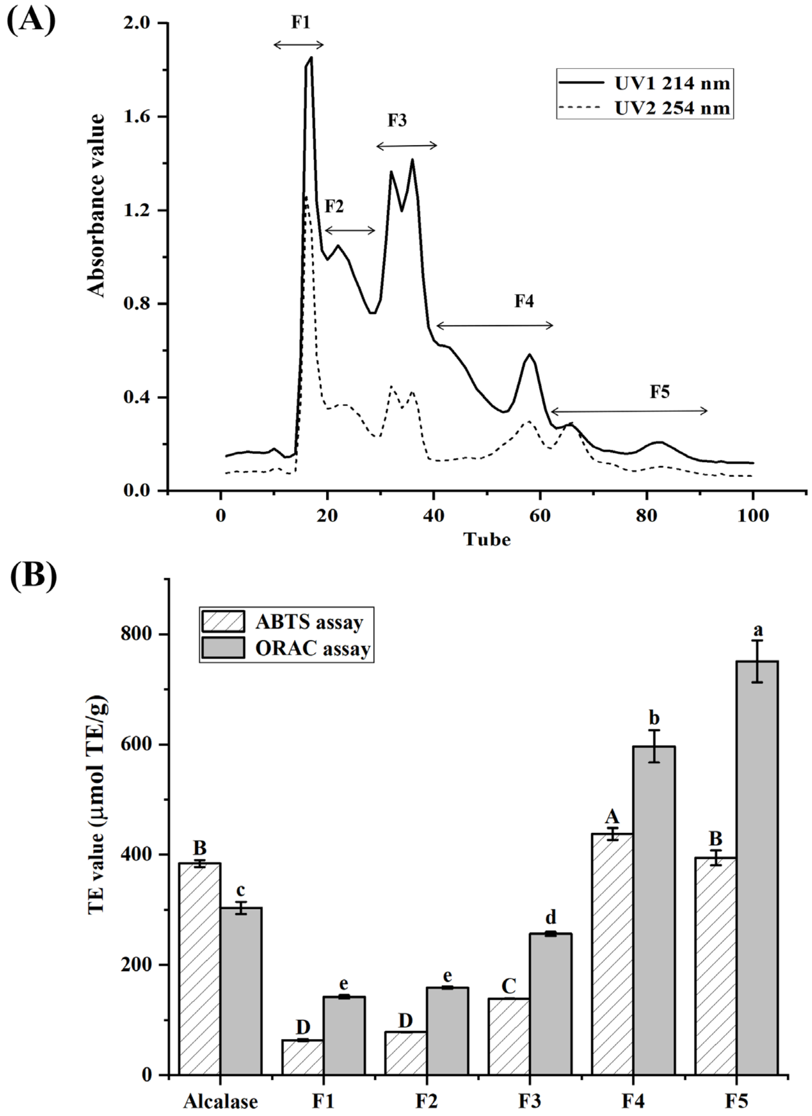

Note: F1 (fractions collected from tubes 12–20), F2 (tubes 21–28), F3 (tubes 29–40), F4 (tubes 41–62), and F5 (tubes 63–90) represent the five separated fractions.

Figure S3 Identification of antioxidant peptides NVGW and WALN from F4 fraction utilizing LC–MS/MS

(A)

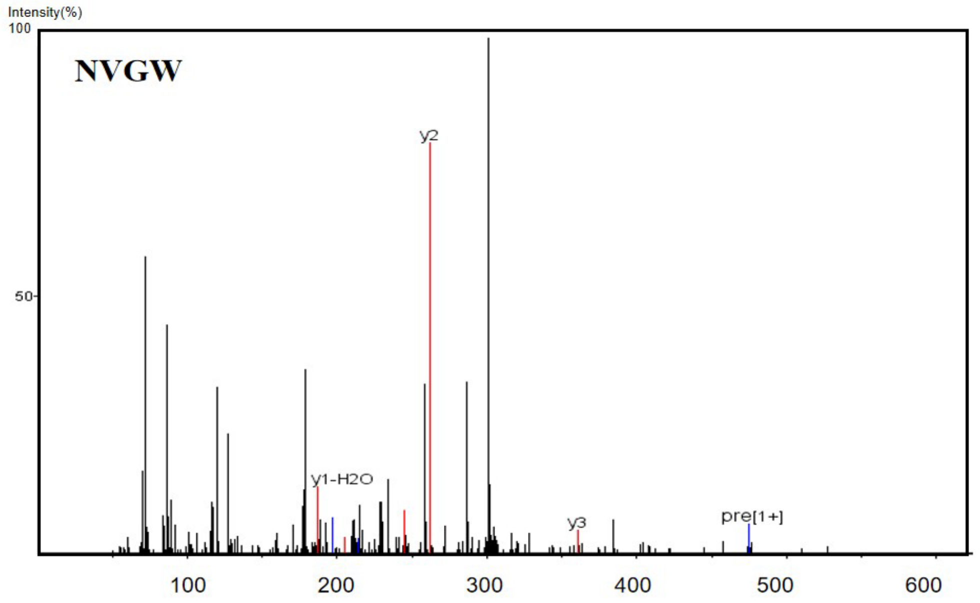

(B)

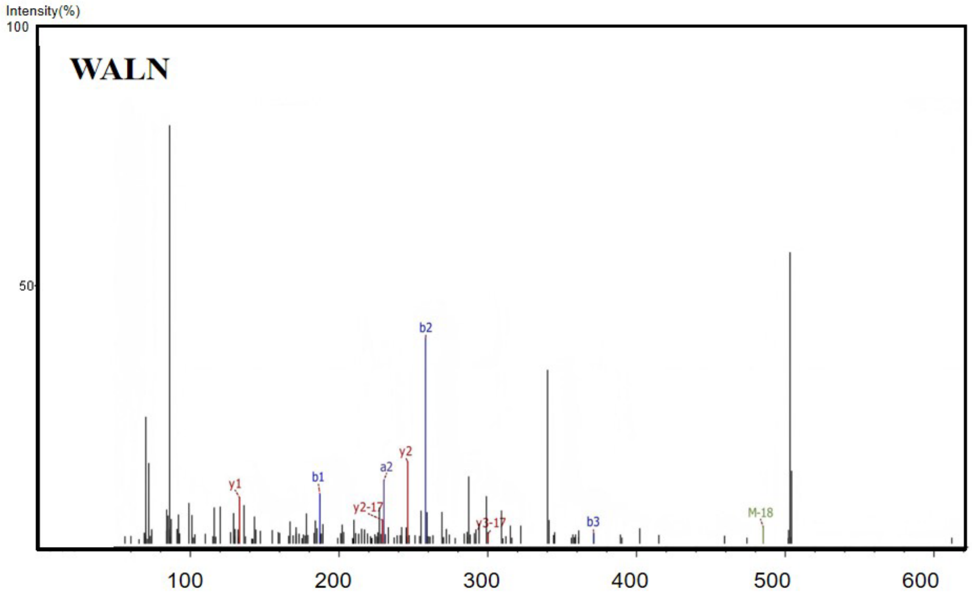

Figure S4 Effects of antioxidant peptides on mortality (A-C) and deformation (D-F) in zebrafish embryos.

Zebrafish embryos were exposed to antioxidant peptide solutions at concentrations of 0, 50, 100, 200, 400, and 800 μM at 8 hours post-fertilization (8 hpf) and were subsequently cultured for 120 hours, with medium changes and cleaning performed every 24 hours. Mortality and deformation were assessed between days 1 and 5 for embryos subjected to different peptide concentrations, and the results are presented in Figure S4. Zero mortality and deformation were observed in the control group after 5 days, confirming the validity of the experimental conditions. At 200 μM, mortality was observed in four peptide groups. Among these, DFLRY and WDAAPGVPR exhibited lower mortality, ranging from 0–20% ± 5.78%, whereas WDPDAR and WGDLSRP showed slightly higher mortality, ranging from 0–43.33% ± 6.67%. Mortality further increased at 400 μM. At 800 μM, DFLRY remained associated with high mortality, reaching 100%, while mortality in the other three peptides was not observed. WGKLD, RWPVDL, GPYLDLK, and WGDLSPK demonstrated mortality at both 400 and 800 μM, with mortality increasing at higher concentrations. Mortality was observed for WLDQPH only at 400 μM, whereas NVGW, WALN, GYLPR, YVDRF, and RDWPDAR exhibited mortality exclusively at 800 μM. Exposure to 200 μM WGKLD resulted in deformation after 2 days, which were aggravated at 400 μM, while no significant deformation was detected at 800 μM. WALN and RDWPDAR exhibited deformation at 400 and 800 μM, which decreased at higher concentrations. Deformation was observed for WLDQPH and RWPVDL only at 400 and 800 μM, respectively.

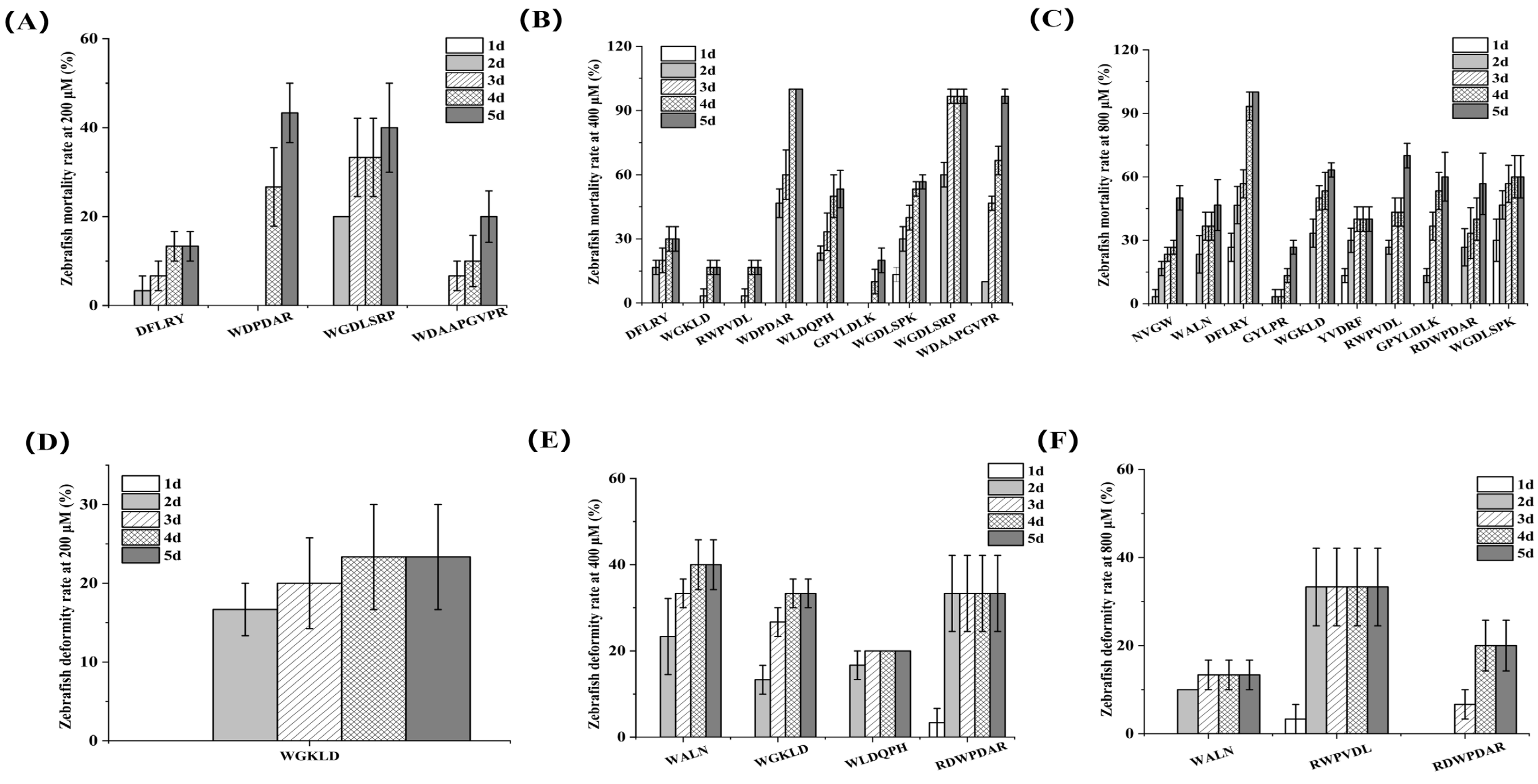
(A)
Zebrafish mortality rate at 200 μM (%)
1d
2d
3d
4d
5d
DFLRY
WDPDAR
WGDLSRP
WDAAPGVPR
(B)
Zebrafish mortality rate at 400 μM (%)
DFLRY
WGKLD
RWPVDL
WDPDAR
WLDQPH
GPYLDLK
WGDLSPK
WGDLSRP
WDAAPGVPR
(C)
Zebrafish mortality rate at 800 μM (%)
NVGW
WALN
DFLRY
GYLPR
WGKLD
YVDRF
RWPVDL
GPYLDLK
RDWPDAR
WGDLSPK
(D)
Zebrafish deformity rate at 200 μM (%)
WGKLD
(E)
Zebrafish deformity rate at 400 μM (%)
WALN
WGKLD
WLDQPH
RDWPDAR
(F)
Zebrafish deformity rate at 800 μM (%)
WALN
RWPVDL
RDWPDAR
